# Inhibiting CSF1R alleviates cerebrovascular white matter disease and cognitive impairment

**DOI:** 10.1002/glia.24481

**Published:** 2023-11-01

**Authors:** Katharine E. Askew, Joshua Beverley, Emma Sigfridsson, Stefan Szymkowiak, Katherine Emelianova, Owen Dando, Giles E. Hardingham, Jessica Duncombe, Edel Hennessy, Juraj Koudelka, Neshika Samarasekera, Rustam Al‐Shahi Salman, Colin Smith, Adriana A. S. Tavares, Diego Gomez‐Nicola, Raj N. Kalaria, Barry W. McColl, Karen Horsburgh

**Affiliations:** ^1^ Centre for Discovery Brain Sciences University of Edinburgh Edinburgh UK; ^2^ UK Dementia Research Institute University of Edinburgh Edinburgh UK; ^3^ Centre for Clinical Brain Sciences and Sudden Death Brain Bank University of Edinburgh Edinburgh UK; ^4^ British Heart Foundation Centre for Cardiovascular Science University of Edinburgh Edinburgh UK; ^5^ School of Biological Sciences University of Southampton Southampton UK; ^6^ Clinical and Translational Research Institute Newcastle University Newcastle UK

**Keywords:** cerebrovascular disease, CSF1R, hypoperfusion, microglia, vascular cognitive impairment, white matter

## Abstract

White matter abnormalities, related to poor cerebral perfusion, are a core feature of small vessel cerebrovascular disease, and critical determinants of vascular cognitive impairment and dementia. Despite this importance there is a lack of treatment options. Proliferation of microglia producing an expanded, reactive population and associated neuroinflammatory alterations have been implicated in the onset and progression of cerebrovascular white matter disease, in patients and in animal models, suggesting that targeting microglial proliferation may exert protection. Colony‐stimulating factor‐1 receptor (CSF1R) is a key regulator of microglial proliferation. We found that the expression of *CSF1R/Csf1r* and other markers indicative of increased microglial abundance are significantly elevated in damaged white matter in human cerebrovascular disease and in a clinically relevant mouse model of chronic cerebral hypoperfusion and vascular cognitive impairment. Using the mouse model, we investigated long‐term pharmacological CSF1R inhibition, via GW2580, and demonstrated that the expansion of microglial numbers in chronic hypoperfused white matter is prevented. Transcriptomic analysis of hypoperfused white matter tissue showed enrichment of microglial and inflammatory gene sets, including phagocytic genes that were the predominant expression modules modified by CSF1R inhibition. Further, CSF1R inhibition attenuated hypoperfusion‐induced white matter pathology and rescued spatial learning impairments and to a lesser extent cognitive flexibility. Overall, this work suggests that inhibition of CSF1R and microglial proliferation mediates protection against chronic cerebrovascular white matter pathology and cognitive deficits. Our study nominates CSF1R as a target for the treatment of vascular cognitive disorders with broader implications for treatment of other chronic white matter diseases.

## Main Points

- CSF1R inhibition a) prevents microglial proliferation in chronic hypoperfused white matter and b) reduces white matter pathology and improves cognitive abilities.
- CSF1R proposed as a target to treat vascular cognitive disorders.

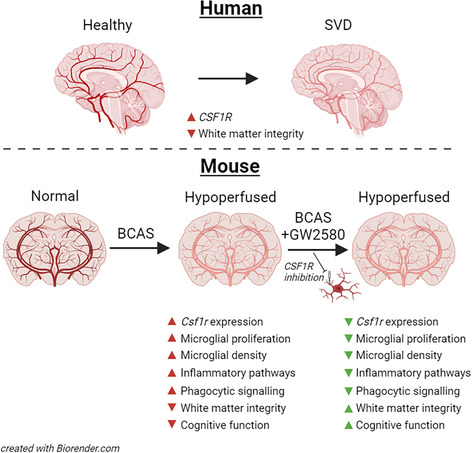

## 1 INTRODUCTION

White matter abnormalities, caused by hypoperfusion/ischemia, are a core feature of small vessel disease (SVD), the most common cerebrovascular disorder leading to cognitive impairment (Hase et al., 2018). Neuroimaging approaches, such as fluid‐attenuated inversion recovery (FLAIR) or T_2_‐weighted magnetic resonance imaging (MRI) sequences, identify white matter abnormalities as white matter hyperintensities (WMHs). The extent and presence of WMHs are clinically important; they increase with age and are predictive of cognitive function, dementia onset and progression (Prins & Scheltens, 2015; Wardlaw et al., 2019). Identification of the earliest molecular and cellular mechanisms leading to white matter damage and cognitive dysfunction is of critical importance to identify therapeutic targets that reduce the onset and/or progression of cognitive decline and dementia.

Accumulating evidence implicates microglia and neuroinflammatory mechanisms in cerebrovascular‐mediated white matter dysfunction. Neuropathological evaluation of WMHs and lesions show loss of myelin and axonal degeneration, which is accompanied by robust microvascular inflammation and gliosis, including increased numbers of reactive microglia (Al‐Mashhadi et al., 2015; Hase et al., 2018; Simpson et al., 2007; Simpson et al., 2007; Simpson et al., 2009; Waller et al., 2020; Wardlaw et al., 2019; Wharton et al., 2015). Further support for the involvement of inflammatory and immunological processes in white matter pathology has been demonstrated by recent genome‐wide association studies (GWAS) of cerebrovascular disease (Armstrong et al., 2020; Persyn et al., 2020). These studies linked variants in genes (e.g. *HLA‐B*, *HLA‐S*, *NMT1*, *NEURL1*, *UNC13D*), that have enriched or specific expression in microglia and macrophages, to neuroimaging markers of white matter abnormalities (Armstrong et al., 2020; Persyn et al., 2020) and highlight the clinical importance of understanding the contribution of neuroinflammatory processes to white matter pathology. Investigations in preclinical models of cerebrovascular disease, including our own, have consistently shown associations between glial reactivity, inflammation and diffuse white matter disease (Coltman et al., 2011; Fowler et al., 2018; Hase et al., 2018; Holland et al., 2011; Jalal et al., 2012; Manso et al., 2018; Rajani et al., 2018; Reimer et al., 2011). Evidence of hypoxia, oxidative stress, microvascular damage, endothelial dysfunction, and blood brain barrier (BBB) disruption have been demonstrated, and notably can be important triggers of glial reactivity and neuroinflammatory responses (Duncombe et al., 2017). These studies have also provided compelling support that inflammation causes white matter disease (Fowler et al., 2018; Jalal et al., 2012; Kaiser et al., 2014; Manso et al., 2018). In particular, this is demonstrated by amelioration of white matter damage following the use of anti‐inflammatory drugs (Fowler et al., 2018; Jalal et al., 2012; Manso et al., 2018).

Microglia exert a major influence on the neuroinflammatory environment in brain disease (Voet et al., 2019). As the main resident macrophages of the CNS, they are important in homeostasis, including normal maintenance of myelin within white matter (Hagemeyer et al., 2017; McNamara et al., 2023). The degenerative ‘microgliopathies’, resulting from mutations in microglial‐enriched genes (e.g., *TREM2*, *TYROBP*, *CSF1R*, *NRROS*) are notable for predominantly affecting white matter (Oosterhof et al., 2019; Paloneva et al., 2002; Smith et al., 2020). These studies highlight the particular importance of microglia to white matter health and, mechanistically, alterations in microglial function could directly cause or potentiate white matter disease progression, although this is yet to be defined in cerebrovascular disease. In the steady state, microglial proliferation is closely coupled to apoptosis to maintain microglial density and homeostasis throughout the lifetime (Askew et al., 2017), but this is disrupted in degenerative conditions resulting in aberrant microglial proliferation (Gomez‐Nicola et al., 2014). Microglial proliferation is predominantly regulated through activation of the receptor tyrosine kinase, colony stimulating factor 1 receptor (CSF1R), by its ligands, colony stimulating factor 1 (CSF‐1), and interleukin‐34 (IL‐34). Pharmacological modulation of CSF1R (using small molecule inhibitors such as GW2580) is a highly effective method to prevent microglial population expansion. Using this approach, key studies have highlighted the critical role of microglial proliferation in the progression of chronic neurodegenerative disease (Dagher et al., 2015; Gomez‐Nicola et al., 2013; Mancuso et al., 2019; Olmos‐Alonso et al., 2016). The ability to specifically modify microglial expansion in disease, without affecting the survival of resident microglial populations, arguably offers a more clinically relevant approach in disease treatment.

In the present study, we show expression of *CSF1R/Csf1r* and indices of microglia abundance are significantly elevated in damaged white matter in human SVD and in a mouse model of chronic cerebral hypoperfusion. We show that expansion of the microglial population through proliferation in hypoperfused white matter is blocked by CSF1R inhibition (via GW2580). Transcriptomic analysis of hypoperfused white matter showed enrichment of microglial and inflammatory gene sets, including phagocytic genes, and that these were the predominant expression modules modified by CSF1R inhibition. CSF1R inhibition was also determined to significantly alleviate white matter damage, and improved spatial learning acquisition and cognitive flexibility.

## 2 MATERIALS AND METHODS

### 2.1 Post‐mortem human brain tissue

Human brain tissues were obtained from the Medical Research Council Edinburgh Brain Bank within the Lothian study of INtraCerebral Hemorrhage, Pathology, Imaging and Neurological Outcome (LINCHPIN) (Samarasekera et al., 2015) and the Lothian Birth Cohort 1936 (LBC1936) (Deary et al., 2007). Control cases were obtained from sudden, unexpected, non‐suspicious deaths with no known neurological disease in life. For quantitative PCR (qPCR), basal ganglia samples were obtained from cases in which small vessel disease (SVD) burden was assessed as mild (grade 1), moderate (grade 2) or severe (grade 3) (*n* = 18; 53–86 years‐old; 11 men and 7 women), meeting at least the mild criteria for vascular cognitive impairment (Skrobot et al., 2018). Control samples presented no pathological evidence of SVD (grade 0) (*n* = 10; 34–40 years‐old; 7 men and 3 women). Clinical characteristics are summarized in Table 1. Samples were collected at autopsy within 5 days from death, snap frozen in liquid nitrogen and stored at −80°C for further analysis. An adjacent piece of tissue from each case was fixed in 10% formalin for pathological analysis. RNA extraction, cDNA synthesis and qPCR were performed as described below.

**TABLE 1:** Clinical characteristics of human SVD cases and controls included in study

Case ID	Gender	Age		Clinical history				Post‐mortem interval (hours)
			SVD severity grading	Dementia status (at time of death)	Hypertension	Ischaemic stroke	Intracerebral hemorrhage (location; interval to death [days])	
SD008/19	Male	82	3	No	Yes	No	N/A	40
SD016/18	Female	86	3	Dementia	Yes	No	Yes (cerebellar; 2400)	114
SD037/16	Female	84	3	No	Yes	No	Yes (right thalamus; 1355)	105
SD011/16	Male	76	3	Dementia	Yes	No	Yes (left frontal lobe; 4)	109
SD003/16	Male	70	3	Dementia	Yes	Ischaemic stroke	Yes (left parietal lobe; 8)	82
SD017/16	Female	80	2	No	Yes	No	N/A	72
SD025/16	Male	80	2	No	No	No	N/A	57
SD055/12	Male	76	2	No	No	No	N/A	90
SD030/12	Female	71	2	No	Yes	No	N/A	41
SD034/15	Male	69	1	No	No	No	N/A	49
SD039/05	Female	79	1	No	Yes	No	N/A	100
SD036/12	Male	75	1	No	No	No	N/A	78
SD001/11	Male	74	1	No	No	No	N/A	46
SD015/12	Male	70	1	No	Yes	No	N/A	74
SD046/17	Female	65	1	No	Yes	No	N/A	76
SD040/16	Male	57	1	No	No	No	N/A	113
SD028/18	Female	53	1	No	No	No	N/A	107
SD030/18	Male	63	1	No	No	No	N/A	115
SD012/10	Male	40	0	No	No	No	N/A	48
SD010/10	Male	39	0	No	No	No	N/A	81
SD002/10	Male	38	0	No	No	No	N/A	49
SD006/06	Male	40	0	No	No	No	N/A	48
SD026/16	Female	37	0	No	No	No	N/A	126
SD031/15	Female	40	0	No	No	No	N/A	89
SD025/06	Female	40	0	No	No	No	N/A	46
SD038/06	Male	37	0	No	No	No	N/A	41
SD038/17	Male	34	0	No	No	No	N/A	99
SD023/11	Male	36	0	No	No	No	N/A	50

### 2.2 Animals and surgical procedures

All animal experiments were conducted in accordance with the Animal (Scientific Procedures) Act 1986 and local ethical approval at the University of Edinburgh and were performed under personal and project licenses granted by the Home Office.

Adult (25‐30 g) male C57BL/6J mice were purchased from Charles River Laboratories. Mice underwent bilateral carotid artery stenosis (BCAS) as we previously described (Coltman et al., 2011). Both common carotid arteries were isolated under isoflurane anesthesia and microcoils (0.16 mm and 0.18 mm internal diameter, Sawane Spring Co, Shizuoka, Japan) placed permanently on the left and right common carotid arteries, respectively. A 30‐minute interval was left between the placement of the first and second coil. Sham animals underwent the same procedure except the placement of the microcoils.

Baseline measurements of cortical cerebral perfusion were acquired 24 hours prior to BCAS or sham surgery using a Moor FLPI2 laser speckle contrast imager (Moor Instruments). Further measurements were taken 24 hours and 6 weeks following surgery. Animals were anesthetized using isoflurane and restrained on a stereotactic frame. Body temperature was monitored throughout and maintained at 37+/− 0.5°C using a heat pad. The skull was exposed by a midline incision and reflection of the skin of the head. The exposed skull was covered with a water‐based gel and cortical perfusion measures recorded. Following recording, the skin was sutured, and a local anesthetic applied. Animals were recovered in a temperature‐regulated box prior to return to home cage. Stable blood flow recordings in the barrel cortex for 2 min was used for analysis. Speckle contrast images were analyzed using MoorFLPI‐2 Review software (version 4.0).

### 2.3 Experimental design

Experiments were designed in accordance with the Animal Research: Reporting of In Vivo Experiments (ARRIVE) 2.0 guidelines. Animals were randomly assigned to surgery and drug treatment groups. Experimenters were blinded to the surgery and drug treatment status of the mice throughout data collection and analysis. Mice were carefully monitored throughout all experimental procedures. To assess the short‐term effects of hypoperfusion on microglial proliferation, mice were allocated to sham or BCAS surgery to be studied 1 week after surgery. In order to assess the longer‐term chronic effects of hypoperfusion on microglial proliferation, white matter pathology and cognition, mice were allocated to sham or BCAS surgery and treatment with either vehicle sham, vehicle BCAS or GW2580 BCAS and studied 6 weeks after surgery. The majority of mice recovered well, but those that did not were excluded from the study (*n* = 6 excluded in 1 week and *n* = 7 excluded in 6‐week studies due to poor recovery). All mice that were excluded were in vehicle treated hypoperfused group. The final cohort sizes were as follows: short‐term study (7 day pathology) – sham *n =* 13, BCAS *n =* 14; chronic study (6 week pathology and behaviour) – sham *n =* 9, BCAS *n =* 12, BCAS + GW2580 *n =* 10. A subset of the mice from the chronic study were used for bulk RNA sequencing ‐ *n* = 8/group. An additional cohort was used for FACS and qPCR analysis at 7 days ‐ sham *n* = 3, BCAS *n* = 6.

### 2.4 Drug administration

To evaluate the effects of GW2580 (LC Laboratories, PKC Pharmaceuticals Inc.) following 6 weeks BCAS, mice were fed with a control diet (RM1) or a diet containing GW2580 [Modified LabDiet® PicoLab EURodent Diet 14%, 5L0W (5LF2) with 0.1% (1000 ppm) GW2580 (LC Laboratories); TestDiet] for 6 weeks, beginning 24 hours post‐surgery.

For the evaluation of microglial proliferation, mice were dosed orally with 5‐bromo‐2′‐deoxyuridine (BrdU; 50 mg/kg in 0.5% Hypromellose and 0.1% Tween80; Sigma Aldrich) for 3 consecutive days prior to sacrifice.

### 2.5 Barnes maze to assess behavioral alterations

Cognitive alterations were assessed using the Barnes maze at 6 weeks following BCAS or sham surgery. All experiments were performed in a behavior testing room maintained at a constant temperature of 20°C. The maze consisted of one white circular platform with 20 circular holes around the outside edge, with 91.5 cm diameter and 115 cm height (San Diego Instruments). Lamps and overhead lights (450 lux) were used to light the maze in addition to an aversive white noise stimulus played at 85 dB. A dark escape chamber was attached to one of the holes allocated to each experimental animal. Visual cues were present on the curtains and walls around the maze. Animals were retained within a white holding cylinder (diameter 10.5 cm) at the beginning of each trial. All trials were recorded by video‐based automated tracking system ANY‐Maze v4.99 (Stoelting Europe).

#### 2.5.1 Acclimation and habituation

Animals were brought into the testing room and placed in the holding cylinder to acclimate to the testing environment for 10 seconds for 2 days before habituation. Mice were habituated to the maze and escape chamber 1 day prior to the start of acquisition training. Each mouse was placed in the holding cylinder for 10 seconds then allowed to freely explore the maze with no aversive stimuli for 3 minutes. Mice were then guided to the escape chamber and retained inside for 1 minute. The maze and escape chamber were cleaned with ethanol between each trial to avoid carryover of olfactory cues between animals.

#### 2.5.2 Visuo‐spatial learning and working memory test (acquisition training)

During the acquisition training, mice were trained to locate the escape chamber over 6 consecutive days with 2 trials a day (60‐minute inter‐trial interval). Each mouse was allocated 1 of 20 escape holes and the location of the escape chamber remained constant for each mouse but was shifted 90 degrees clockwise between consecutive mice to avoid carryover of olfactory cues. Mice were retained in the holding cylinder for 10 seconds at the start of each trail. Once the trial started, the aversive noise stimulus (85 dB) was started and switched off once the mouse entered the escape chamber. If the mouse failed to enter the escape chamber during the 3‐minute trial period, the experimenter guided it to the chamber.

#### 2.5.3 Reversal training (cognitive flexibility)

During the reversal training, mice were trained to locate the escape chamber following the same procedure as the acquisition training, however the allocated escape chamber was shifted 180° to the opposite side of the maze. Mice were trained over 3 consecutive days with 2 trials a day (60‐minute inter‐trial interval) in a task with increased difficulty to provide a measure of cognitive flexibility.

#### 2.5.4 Measurements

All trials were recorded by a camera located above the maze and measured using tracking software ANY‐Maze v4.99. Spatial learning was assessed by the total time taken to enter the escape chamber in each trial (escape latency). Total distance traveled and speed during the trials were additionally measured. Exclusion criteria were defined prior to data analysis as follows: mice must enter a minimum of three quadrants of the maze within two of the first five trials. All mice met this criterion and were included for analysis.

### 2.6 Tissue processing

To collect tissue for histology and RNA extraction, mice were sacrificed by cervical dislocation. The brain was removed and transferred to ACSF. A 2 mm slice of the forebrain was taken from which the corpus callosum was dissected out using a dissecting microscope and snap frozen in liquid nitrogen. A 1.6 mm coronal slice was taken at −1.65 mm posterior of bregma and post‐fixed in 4% paraformaldehyde for 24 hours, prior to further processing for paraffin embedding. Paraffin‐embedded tissues were cut into 6 μm coronal sections at −1.70 mm posterior of bregma using a rotary microtome (Leica Microsystems) and mounted onto Superfrost Plus slides (VWR International).

For flow cytometry experiments, mice were transcardially perfused with ice cold PBS with 0.1% heparin under deep isoflurane anesthesia. The brain was removed and quickly cut into 2 mm sagittal slices using a matrix, then transferred to ice cold 1X HBSS with 25 mM HEPES. The corpus callosum and cortex were dissected out using a dissecting microscope and transferred to fresh tubes containing ice cold 1X HBSS with 25 mM HEPES and kept on ice prior to processing.

### 2.7 Immunohistochemistry

Paraffin‐embedded tissue sections were deparaffinized for 30 minutes at 60°C followed by 2 × 15‐minute incubations in xylene. Sections were then rehydrated through a series of alcohols (100%, 90%, 70%) and washed with running water. For DAB (3,3′‐Diaminobenzidine) immunohistochemistry (myelin‐associated glycoprotein [MAG], ionized calcium binding adaptor molecule 1 [Iba1]), sections were quenched in 0.3% H_2_O_2_ in methanol for 30 minutes then washed with running water. Citric acid retrieval (10 mM, pH 6.0) was carried out at 95°C for 10 minutes in a Decloaking chamber (Biocare Medical). Sections were washed repeatedly in PBS and blocked with 10% normal serum and 0.5% (MAG) or 5% (Iba1) bovine serum albumin (BSA) at room temperature for 1 hour before primary antibody incubation overnight at 4°C. Biotinylated secondary antibodies were incubated for 1 hour at room temperature and then further amplified; 1 hour at room temperature in Vector ABC Elite Kit (Vector, UK), before visualization of peroxidase activity using DAB (Vector Labs, UK). Sections were then washed in running water and dehydrated through a series of alcohols (70%, 90%, 100%) to xylene and then mounted using DPX (Sigma, UK). For immunofluorescence (Iba1, BrdU [5‐Bromo‐2´‐Deoxyuridine]), primary antibodies were diluted in blocking solution made up with 0.3% TritonX‐100 in PBS prior to overnight incubation. Sections were washed with PBS and incubated with the appropriate biotinylated secondary antibody for 1 hour at room temperature. Following further PBS washes, sections were incubated with AlexaFluor‐conjugated secondary antibodies and streptavidin for 1 hour at room temperature. Sections were then washed with PBS followed by Tris buffer and allowed to partially air‐dry prior to mounting with Vectashield Hardset Mounting Medium with DAPI (Vector, UK). A modified immunofluorescence protocol with tyramide signal amplification was used for Iba1^+^/Lamp2^+^ (lysosome‐associated membrane protein2) double staining. Following deparaffinization and rehydration as previously described, antigen retrieval was carried out at 97.5°C using preheated Tris‐EDTA (10 mM Tris buffer, 1 mM EDTA, pH 9.0) for 30 minutes. Following repeated PBS washes, sections were blocked with 10% normal serum for 1 hour before overnight incubation with the first primary antibody (Iba1), 1% BSA and 0.3% Triton X‐100 in PBS. Sections were repeatedly washed in Tris‐buffered saline with 0.1% Tween20 (TBST 0.1%) and quenched in 0.3% H_2_O_2_ in methanol for 30 minutes then washed repeatedly with TBST0.1%. Biotinylated secondary antibodies were incubated for 1 hour at room temperature and then further amplified using the AlexaFluor™ 488 Tyramide SuperBoost™ Kit, following the manufacturer's instructions. Once the tyramide reaction had been developed, sections were washed repeatedly in PBS, re‐retrieved in Tris‐EDTA buffer and re‐blocked in 10% normal serum prior to incubation with the second primary antibody (Lamp2). Visualization of Lamp2 was performed using standard Alexa Fluor‐conjugated secondary antibodies as described previously. Primary antibodies and concentration were as follows; Iba1 rabbit polyclonal Menarini, cat no. MP‐290, 1:500 (immunofluorescence); Iba1 rabbit polyclonal Wako, cat no. 019–19,741, 1:500 (DAB); BrdU rat monoclonal Abcam, cat no. ab6326, 1:50; MAG mouse monoclonal Abcam, cat no. ab89780, 1:15,000; Ki67 rat monoclonal, eBioscience, cat no. 14–5698‐82, 1:25, Lamp2 rat monoclonal, Biolegend, cat. no. 108501, 1:100.

### 2.8 Analysis of immunohistochemistry

Immunofluorescent‐labeled sections were imaged using an LSM 710 (Zeiss) or TCS‐SP5 confocal microscope (Leica Microsystems). DAB‐labeled sections were imaged using a BX51 bright field microscope (Olympus) or an Axio Scan.Z1 slide scanner (Zeiss). All image analysis was performed using ImageJ software (v1.46, NIH, Bethesda, MD, USA). Microglia were identified using Iba‐1 and the number of positively stained cells in the corpus callosum, internal capsule and fimbria were counted. To evaluate proliferation of microglia, Iba‐1 was co‐labeled with BrdU or Ki67 and the number of Iba‐1^+^/BrdU^+^ or Iba‐1^+^/Ki67^+^ cells were manually counted within the corpus callosum and fimbria. To evaluate microglial phagocytic activity, Iba1 was co‐labeled with Lamp2 and the number of Iba1^+^/Lamp2^+^ cells was manually counted within the corpus callosum. Iba1^+^/Lamp2^+^ cell counts were then normalized to the total Iba1^+^ cell count. White matter damage determined by MAG immunostaining was graded from 0 (none) to 3 (extensive). Myelin damage identified with MAG was determined as the presence of disorganized white matter fibers and myelin debris. The scale was as follows; normal (grade 0), minimal myelin debris, vacuolation, and disorganization of fibers (grade 1), modest myelin debris, vacuolation, and disorganization of fibers (grade 2), and extensive myelin debris, vacuolation, and disorganization of fibers (grade 3).

### 2.9 Flow cytometry and cell sorting

For processing of tissue into a single cell suspension, all tools and reagents were kept ice cold throughout and centrifugation steps were performed at 4°C. Corpus callosum or cortical samples were quickly minced in 1X HBSS (without Ca^2+^ or Mg^2+^; Gibco) with 25 mM HEPES (Fisher Scientific) (HBSS with HEPES) using a scalpel prior to transfer to a 2 mL dounce homogenizer. Tissue samples were homogenized with 30 passes of the dounce then filtered through a pre‐wet 70 μm cell strainer (BD2 Falcon) which was washed with 2 mL HBSS with HEPES. Samples were centrifuged at 600 *x g* for 5 minutes prior to resuspension in a 30% PercollPLUS solution with 5 mL HBSS with HEPES overlaid on top. The cell suspension was then centrifuged at 600 *x g* for 20 minutes with no break. Cells were resuspended in FACS buffer (1X PBS [Gibco] with 25 mM HEPES and 0.1% BSA [Sigma Aldrich]) and incubated with Mouse BD Fc Block™ (BD Biosciences) for 30 minutes on ice. Cells were then immunostained with primary antibodies directed against CD11b (clone: M1/70), CD45 (clone: 30‐F11), Ly6C (clone: HK1.4), Ly6G (clone: 1A8) at 1:200 (Ly6C) or 1:500 dilution for 30 minutes on ice. Cells were washed then analyzed and sorted using a FACS Aria II (BD Biosciences). Single‐stained and buffer only (“unstained”) samples were used as controls. Positively stained populations were defined relative to the unstained control. Cell sorting was performed by staff in the QMRI Flow Cytometry and Cell Sorting Facility (University of Edinburgh). Data were acquired with FACSDiva software (Becton Dickinson). Post‐acquisition analysis was performed using FCS Express 6 software (De Novo Software) and the full gating strategy for analysis is detailed in Supplementary Figure S2a.

### 2.10 RNA extraction and cDNA synthesis

For post‐mortem human brain tissue and mouse tissue (corpus callosum) samples, RNA was extracted using the RNeasy Lipid Tissue Mini Kit (Qiagen) according to the manufacturer's instructions. RNase‐free DNAse I (Thermo Scientific) was used to remove genomic DNA according to the manufacturer's instructions. RNA quantities were determined by Nanodrop 1000 (Thermo Fisher Scientific). For mouse samples, cDNA was synthesized using the Transcriptor First Strand cDNA Synthesis Kit (Roche) according to the manufacturer's instruction. cDNA was then diluted to the equivalent of 3 ng initial RNA per 15 μL qPCR reaction. For human samples, cDNA was synthesized using Superscript IV Reverse Transcriptase (Life Technologies) at a concentration of 15 ng/μl and used undiluted for qPCR reactions. For sorted cells, RNA was extracted using the High Pure RNA Isolation Kit (Roche) according to the manufacturer's instructions. RNA quantities were determined by Agilent 4200 Tapestation (Agilent Technologies). cDNA was synthesized from 1.5 ng RNA using Superscript IV Reverse Transcriptase (Life Technologies), according to the manufacturer's instructions and used undiluted. cDNA samples were stored at −20°C until use.

### 2.11 Quantitative PCR

cDNA libraries were analyzed by qPCR using the DyNAmo ColorFlash SYBR Green kit (Thermo Scientific) according to the manufacturer's instructions. Briefly, the cDNA template was mixed with DyNAmo ColourFlash SYBR Green master mix (Fisher Scientific), nuclease‐free H_2_O and the custom designed gene‐specific primers listed in Supplementary Table 1 (200 nM final concentration; Sigma Aldrich). Primers were validated to confirm efficiency prior to use. qPCR cycles were performed on a Bio‐Rad CFX96 thermocycler (Bio‐Rad Laboratories) as follows: hot‐start denaturation cycle 95°C for 10 min, 40 cycles of amplification at 95°C for 15 s, primer annealing with or without extension at optimized temperatures as specified in Table 2, followed by one cycle of 95°C for 1 min, 55°C for 30 s, with a ramp up to 30 s at 95°C with continuous detection of fluorescence. Cycle threshold (Ct) values of target genes were normalized to *Aif1* for sorted cells or *18S* for tissue samples and data are expressed as fold change relative to control group (sham) using the 2^(−ΔΔCt) method.

### 2.12 RNA sequencing

RNA was extracted from corpus callosum samples as described above. RNA concentration and integrity were assessed using a LabChip GX24 Nucleic Acid Analyzer (Perkin Elmer) using a RIN score minimum threshold of 8. Library preparation and sequencing was performed by Edinburgh Genomics. Barcoded cDNA libraries were prepared using the TAKARA SMARTer Stranded Pico Input Mammalian kit, and pooled libraries were sequenced on one lane of an Illumina NovaSeq instrument. Raw reads were quality checked using MultiQC (version 1.8) (Ewels et al., 2016), ensuring quality metrics such as read mapping quality and rate, and rate of duplicates are were within acceptable bounds. Reads were mapped to the Mus musculus genome (GRCm38) annotated with Ensembl (version 98) using splice aware aligner STAR (version 2.7) (Dobin et al., 2013), and assigned to features using featureCount (version 1.6.3) (Liao et al., 2014).

DESeq2 (Love et al., 2014) (version 1.24.0) was used to identify differentially expressed genes. Comparisons were made between sham vs hypoperfused and hypoperfused vs hypoperfused + GW2580 samples. Gene set analysis was performed using Camera implemented in the Limma package (version 3.40.6), using GO gene sets (collection C5) from version 7.0 of the Molecular Signatures Database, MSigDB (Liberzon et al., 2011). Cell type‐specific gene sets were constructed from the “Brain RNA‐seq” data set (https://www.brainrnaseq.org [Zhang et al., 2014; Zhang et al., 2016]), as sets of genes that were 10‐fold more highly expressed in one cell type than any other, with the exception of myelinating and newly formed oligodendrocytes gene sets, which were 3‐fold more highly expressed.

### 2.13 Statistical analysis

Statistical analysis was performed using SPSS (v22, IBM Corp.) or Graphpad Prism (v5, Graphpad Software Inc.). Data are presented as mean ± SD for continuous data, which were analyzed by unpaired t‐test or one‐way ANOVA. Median with interquartile range are shown for data with skewed distribution, which were analyzed with either Mann Whitney‐U or Kruskal‐Wallis H tests with Dunn's post hoc. Repeated measures ANOVA was used to analyze CBF and spatial learning over time/trials in the Barnes Maze. Bonferroni adjustment was used for post hoc analysis. Correlation analyses were done using a Spearman test. Significance was determined at *p* < .05. All data analysis was done by researchers blinded to the genotype, surgery, and drug administration where appropriate.

## 3 RESULTS

### 3.1 *CSF1R* gene expression is increased in white matter and correlates with burden of pathology in human SVD

**FIGURE 1:**
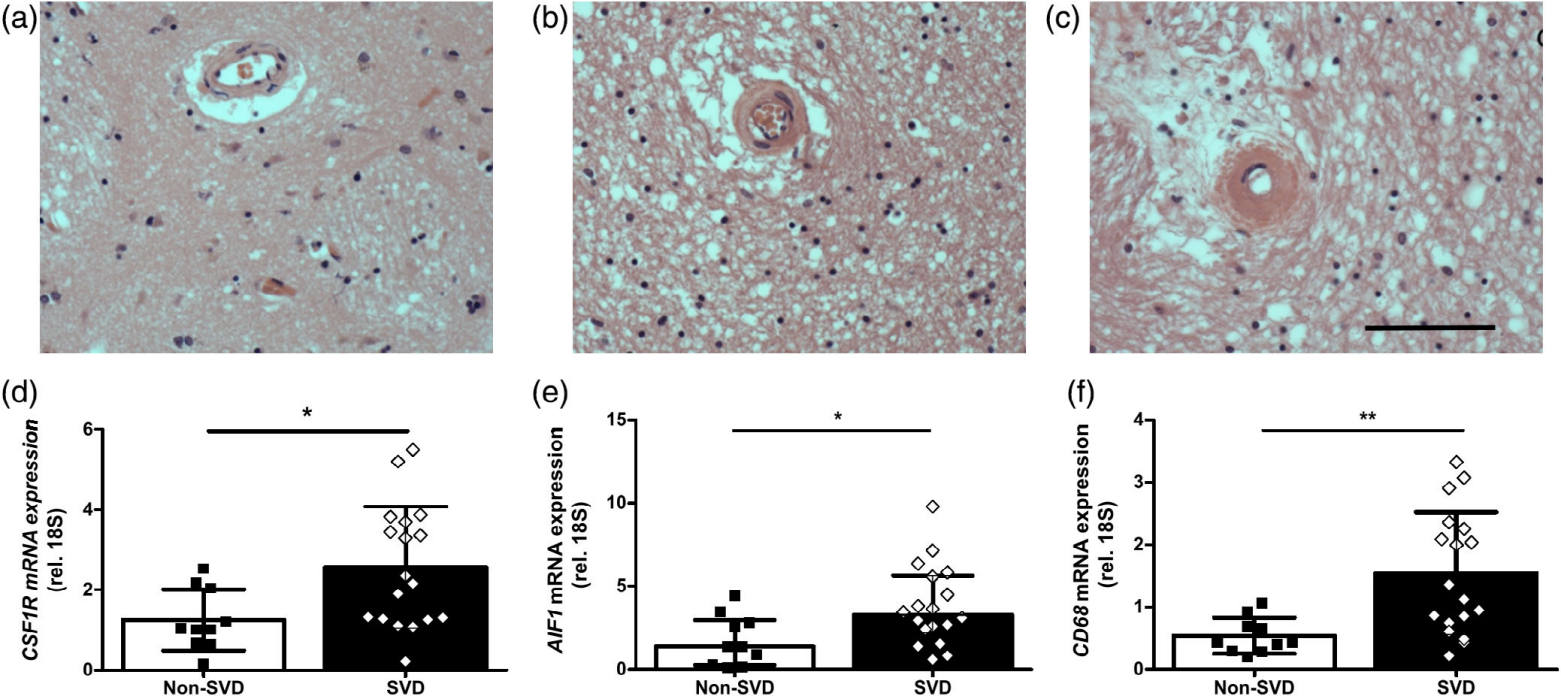
Human SVD is associated with increased *CSF1R* gene expression and microglial activation. (a–c) Hematoxylin and eosin stained representative sections of white matter from basal ganglia of human cases without SVD (a) and with mild–moderate (b) and severe (c) SVD, highlighting white matter arterioles that are (a) normal and (b) with arteriolosclerosis and loss of smooth muscle cells and (c) exhibiting vessel wall hyalinosis and complete loss of smooth muscle cells. (d–f) qPCR analysis of the relative mRNA expression of *CSF1R* (d), *AIF1* (e) and *CD68* (f) in white matter‐enriched basal ganglia tissue samples from SVD patients (*n* = 18) and non‐SVD control cases (*n* = 10). Data presented as mean ± SD and analyzed by Students' *t* test. *p < .05, **p < .01.

Neuroinflammatory mechanisms have been implicated in both histological and transcriptomic analysis of SVD and age‐related white matter disease (Al‐Mashhadi et al., 2015; Armstrong et al., 2020; Hase et al., 2018; Persyn et al., 2020; Simpson et al., 2007; Simpson et al., 2007; Simpson et al., 2009; Waller et al., 2020; Wardlaw et al., 2019; Wharton et al., 2015), however the role of CSF1R signaling has not yet been studied. To investigate this, we studied white matter enriched‐tissue from the basal ganglia from post‐mortem SVD patients, with mild to severe SVD burden, and healthy controls with no SVD pathology (Figure 1a–c; Table 1). In comparison to healthy non‐SVD controls, SVD patients had significantly increased *CSF1R* expression (*p* < .05; Figure 1d), which correlated with SVD burden (Spearman r = 0.4712, *p* < .05), implicating this pro‐mitogenic pathway in white matter pathology. We also found significantly increased expression of *AIF1* (*p* < .05; Figure 1e) and *CD68* (*p* < .01; Figure 1f), suggestive of increased microglial activation in SVD white matter. Both of these markers also significantly correlated with SVD burden (*AIF1* Spearman r = 0.4243, *p* < .05; *CD68* Spearman r = 0.5563, *p* < .01), further implicating microglial activation with white matter disease.

### 3.2 Increased microglial proliferation and *Csf1r* gene expression in a hypoperfusion model of white matter disease

**FIGURE 2:**
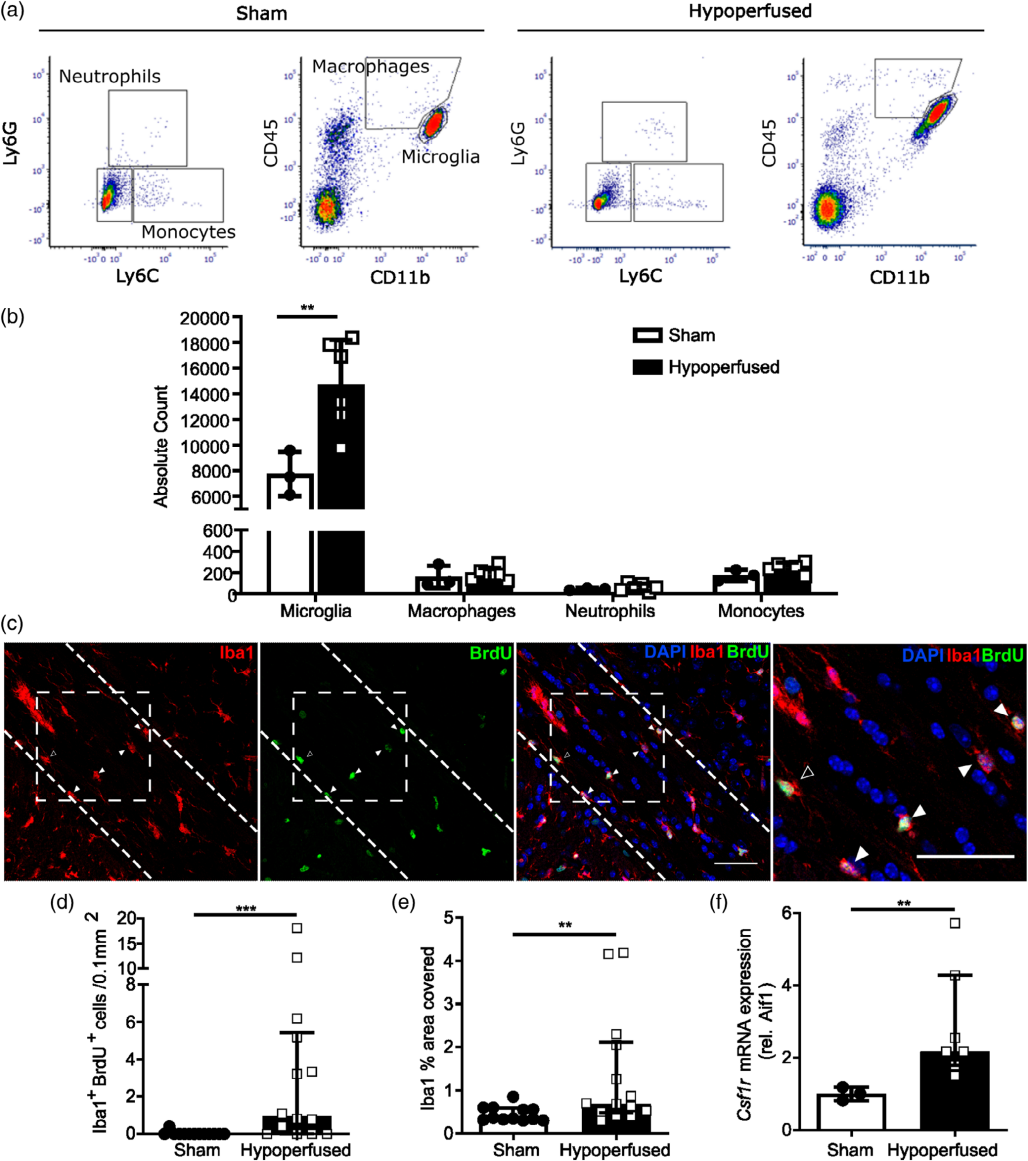
Increased microglial proliferation and *Csf1r* gene expression in a hypoperfusion model of white matter disease (a) Representative flow cytometry dot plots identifying neutrophil (Ly6G^+^), monocyte (Ly6C^+^), microglia (CD11b^+^ CD45^low^ Ly6C^−^ Ly6G^−^) and macrophage (CD11b^+^ CD45^high^ Ly6C^−^ Ly6G^−^) populations in the corpus callosum 7 days post‐surgery. (b) Flow cytometric quantification of the absolute numbers of microglia, macrophages, neutrophils and monocytes in the corpus callosum of sham (*n* = 3) and hypoperfused (*n* = 6) mice, based on the gating strategy shown in (a). Full gating strategy shown in Supplementary Figure 2a. (c) Representative images of Iba1^+^ /BrdU^+^ staining in the corpus callosum. Empty arrowheads indicate BrdU^+^ single positive cells and filled arrowheads represent Iba1^+^/BrdU^+^ cells. Scale bar = 50 μm. (d) Quantification of the number of proliferating microglial cells (Iba1^+^ BrdU^+^) and (e) Quantification of Iba1% area staining in the corpus callosum, 7 days following hypoperfusion in the corpus callosum of sham (*n* = 13) and hypoperfused (*n* = 14) mice. (f) qPCR analysis of the relative mRNA expression of *Csf1r* in FACS‐isolated white matter microglia from sham (*n* = 3) and hypoperfused (*n* = 6) mice. Data presented as mean ± SD and analyzed by Students' *t* test (b), or median ± IQR and analyzed by Mann Whitney U test (d–f). **p < .01, ***p < .001.

White matter pathology is a core feature of cerebrovascular disease and linked to reduced cerebral perfusion (Prins & Scheltens, 2015). In a well‐characterized mouse model of vascular cognitive impairment (VCI) and subcortical white matter disease, we and others have previously demonstrated the vulnerability of white matter tracts to BCAS‐induced hypoperfusion, and shown that this correlates with a robust increase in microglial number (Coltman et al., 2011; Fowler et al., 2018; Holland et al., 2011; Manso et al., 2018; McQueen et al., 2014; Reimer et al., 2011). In this study we wanted to determine whether elevated microglial number is associated with other myeloid cell accumulation in white matter post‐hypoperfusion as these have the potential to contribute to disease progression. Thus, we profiled the myeloid cell response following short‐term (7 days) hypoperfusion using flow cytometric quantification of white and gray matter cell suspensions stained with a panel of myeloid markers (CD11b, CD45, Ly6G, Ly6C), as previously described (Davies et al., 2019) (Figure 2a). Reduced cerebral blood flow (CBF) was confirmed in mice post‐hypoperfusion while remaining unchanged in shams (Supplementary Figure S1a,b). The number of microglia (CD11b^+^ CD45^low^) in the corpus callosum significantly increased post‐hypoperfusion compared to sham (*p* < .01; Figure 2a,b). In contrast, an assessment of myeloid subsets indicated no significant differences in the number of inflammatory monocytes (Ly6C^+^), monocyte‐derived cells (CD11b^+^ CD45^high^), or neutrophils (Ly6G^+^) in the corpus callosum of hypoperfused mice compared to shams (*p* > .05; Figure 2a,b). These changes appeared specific to white matter since we detected no changes in the density of microglia or other myeloid cells in cortical gray matter (Supplementary Figure S2b). These data suggested a largely microglial‐restricted expansion. To determine whether microglial proliferation could explain the increase in Iba1^+^ cells post‐hypoperfusion, we measured the extent of BrdU^+^ incorporation within Iba1^+^ cells (Figure 2c). We found a significant increase in the number of Iba1^+^ BrdU^+^ cells in the corpus callosum compared to sham (*p* < .001; Figure 2d), as well as a significant increase in Iba1 staining density, suggestive of microglial reactivity (*p <* .001; Figure 2e). Thus, collectively these data indicate that expansion of the numbers of resident microglial population occurs primarily in hypoperfused white matter and in the absence of peripheral myeloid cell infiltration.

Since microglial proliferation is predominantly regulated through CSF1R signaling, we next measured *Csf1r* expression in microglia isolated from the corpus callosum by qPCR. At the mRNA level, *Csf1r* expression was significantly increased in white matter microglia in response to short‐term hypoperfusion (*p* < .01; Figure 2f). Together, these data indicate that microglial proliferation, linked to increased expression of *Csf1r*, is a key early change post‐hypoperfusion in white matter.

### 3.3 CSF1R inhibition blocks the expansion of microglia in hypoperfused white matter

**FIGURE 3:**
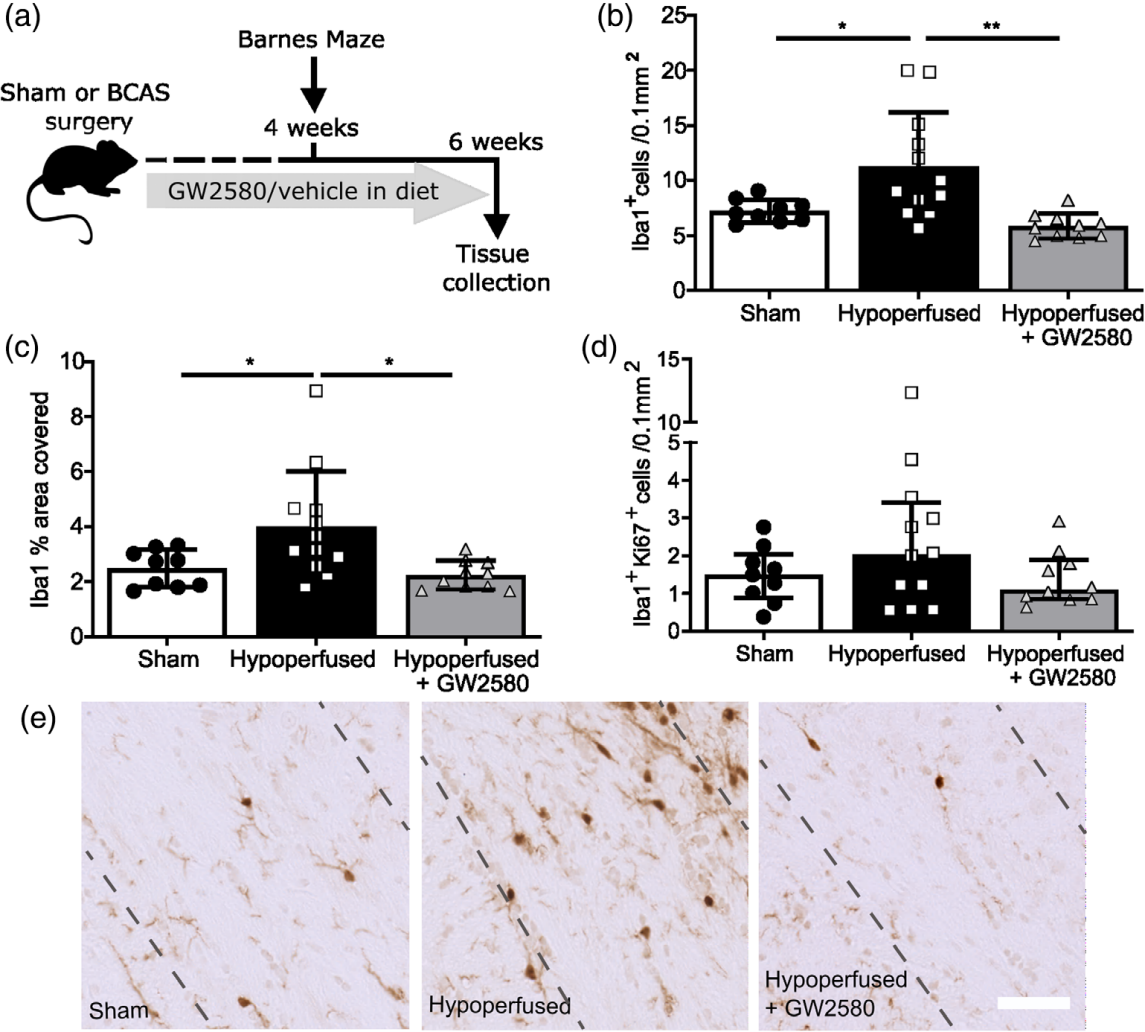
CSF1R inhibition following chronic hypoperfusion prevents expansion of microglia in the white matter. (a) Experimental scheme for the chronic hypoperfusion study. Final n numbers: sham, *n* = 9; hypoperfused + vehicle, *n* = 12; hypoperfused + GW2580, *n* = 10. (b) Quantification of the number of microglial cells (Iba1^+^) and (c) Iba1% area staining as a measure of microglial activation in the corpus callosum following 6 weeks of hypoperfusion and GW2580 treatment. (d) Quantification of the number of proliferating microglial cells (Iba1^+^ Ki67^+^) in the corpus callosum following chronic hypoperfusion and GW2580 treatment. (e) Representative images of Iba1 staining in the corpus callosum. Scale bar = 50 μm. Data presented as mean ± SD and analyzed by one‐way ANOVA with *post hoc* Bonferroni correction (b–d), *p < .05, **p < .01.

To block microglia proliferation in vivo, we utilized GW2580, an orally available, brain‐penetrant, inhibitor of CSF1R, at a dose that inhibits microglial proliferation without affecting cell survival (Gomez‐Nicola et al., 2013; Martinez‐Muriana et al., 2016; Olmos‐Alonso et al., 2016). Post‐hypoperfusion, mice were fed a diet with GW2580 or vehicle and shams fed a vehicle diet over 6 weeks (Figure 3a).

At the outset, we first determined CBF changes to assess whether there was an impact of GW2580 treatment that may influence overall outcome. Cortical CBF was measured using laser speckle imaging at baseline (24 hours before surgery) then at 24 hours and 6 weeks post‐surgery. The extent of CBF reductions post‐BCAS in vehicle‐ and GW2580‐treated mice were determined and compared to shams. Overall, there was a significant effect of time (F_(1.57,43.97)_ = 167.4, *p =* .0001) and BCAS surgery (F_(2,28)_ = 107.2, *p =* .0001) and a significant interaction between time and surgery (F_(3.14,43.97)_ = 30.9, *p =* .0001). Post hoc analysis revealed a significant reduction in CBF 24 hours post‐BCAS in both vehicle‐ and GW2580‐treated mice (*p <* .001), which was persistent at 6 weeks (*p <* .001). GW2580‐treated BCAS mice had similar reductions in CBF to vehicle‐treated BCAS mice (Supplementary Figure S1c,d).

Following 6 weeks of vehicle or drug treatment, microglia numbers, activation and proliferation were evaluated in the corpus callosum (Figure 3b). Overall, there was a significant effect of treatment on the number of Iba1^+^ microglia within the corpus callosum (F_(2,28)_ = 8.89, *p* < .001, Figure 3b), with *post hoc* analysis revealing increased microglial numbers post‐hypoperfusion (11.3 ± 4.9 cells/0.1 mm^2^) compared to sham mice (7.2 ± 1.0 cells/0.1 mm^2^, *p* < .05). Furthermore, treatment with GW2580 significantly reduced microglial numbers post‐hypoperfusion as compared to vehicle‐treated hypoperfused mice (5.9 ± 1.1 cells/0.1 mm^2^, *p* < .001). Similarly, there was an overall effect of treatment on Iba1‐immunoreactive area coverage in the corpus callosum (F_(2,28)_ = 5.73, *p* < .01, Figure 3c) with *post‐hoc* analysis revealing significant increases in Iba1 area coverage post‐hypoperfusion (4.0 ± 2.0%) in comparison to GW2580‐treated hypoperfused mice (2.2 ± 0.5%, *p* < .05), which had a similar level of staining to sham mice (2.5 ± 0.7%, *p* < .01). Similar effects were identified in other white matter regions, with GW2580 treatment significantly reducing microglial numbers in the internal capsule (*p* < .01; Supplementary Figure S3a) and fimbria (*p* < .05; Supplemental Figure 3b) as well as reducing Iba1‐immunoreactive area in these regions (Supplementary Figure S3c,d). Microglial proliferation in the corpus callosum, as assessed by Iba1/Ki67 immunolabeling, was blocked by GW2580 treatment (Figure 3d), with similar effects also observed in the fimbria (Supplementary Figure S3e). Overall, these data indicate that GW2580 treatment prevents microglial proliferation in chronic hypoperfused white matter.

We further determined if there were effects of CSF1R inhibition on the extent of astrogliosis by measuring glial fibrillary acidic protein (GFAP) immunoreactive area in white matter regions following hypoperfusion. However, we found that astrocyte changes were restricted to the corpus callosum and not as widespread as microglial changes. There was a significant overall effect in the corpus callosum (F_(2,28)_ = 6.09, *p* = .006) and *post hoc* tests determined that the hypoperfused vehicle group displayed significant astrogliosis compared to shams (*p* = .01) and the hypoperfused GW2580 group (*p* = .04) (Supplementary Figure S4a). There was no significant difference between groups in the fimbria or the internal capsule (F_(2,28)_=1.81, *p* = 0.18, F_(2,28)_ = 0.68, *p* = .52 respectively) (Supplementary Figure S4b,c).

### 3.4 CSF1R inhibition modifies the immune‐related transcriptome profile of hypoperfused white matter

**FIGURE 4:**
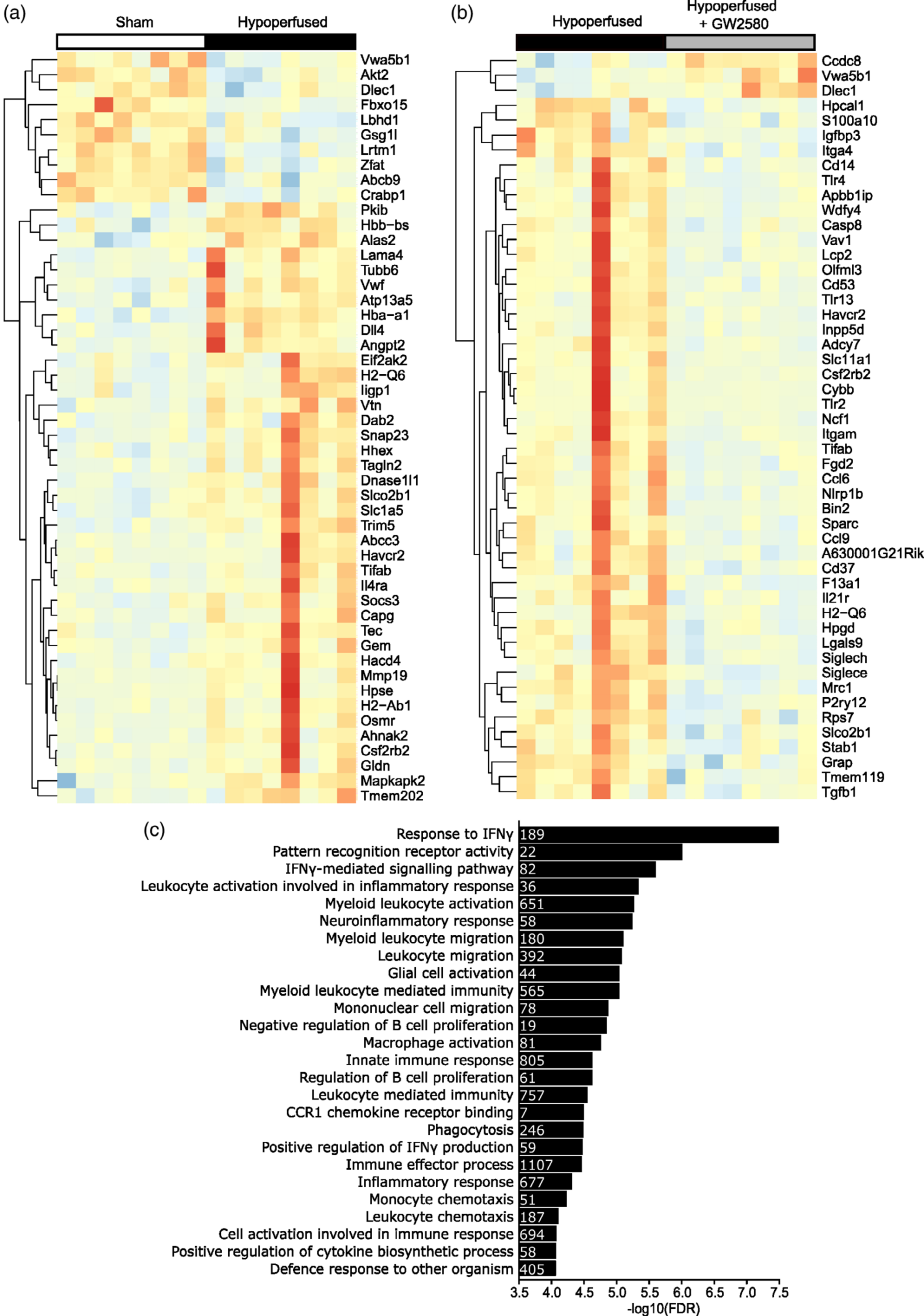
CSF1R inhibition modifies the immune‐related transcriptome profile of hypoperfused white matter (a and b) Heatmaps showing the top 50 most changed genes in white matter samples from sham versus hypoperfused mice (a) and hypoperfused versus hypoperfused + GW2580 mice (b). Data presented as log2‐transformed FPKM and scaled to average Log2 FPKM per gene, red indicating higher and blue lower expression. Sham, *n* = 8; hypoperfused, *n* = 8; hypoperfused + GW2580, *n* = 8. (c) Negative log10 (FDR) for the top gene sets downregulated by GW2580 treatment after chronic hypoperfusion. Inset numbers within bars represent the numbers of genes altered within that pathway.

We next sought to characterize the transcriptomic profile of white matter to identify potential molecular events underlying the effects of CSF1R inhibition in chronic hypoperfused white matter. Using RNA sequencing, we performed a comparative transcriptome profiling analysis of white matter‐enriched samples from sham, vehicle‐hypoperfused and GW2580‐treated hypoperfused mice (*n* = 8/group), from a subset of mice that had previously undergone behavioral and pathology assessment. Of the 35,354 genes identified, differential gene expression analysis determined only one significantly downregulated gene following GW2580 treatment compared to hypoperfusion, *Mrc1* (encoding Mannose Receptor C Type I; CD206, FDR = 5.17 *x* 10^−5^, *p* = 1.44 *x* 10^−9^, Supplementary Table S2). Further investigation of the most altered genes (top 50) between sham and hypoperfused white matter indicated that the majority were increased (Figure 4a, Supplementary Table S2) and related to microvascular inflammation and vascular plasticity (e.g., *Il4ra*, *Angpt2*, *vWF*, *Osmr*, and *Dll4)*. There was also overlap with genes we had previously identified to be altered in a microarray study of white matter 72 hr post‐hypoperfusion (Reimer et al., 2011) (Figure 4a). The majority of genes (94%) were reduced with GW2580 treatment post‐hypoperfusion compared to vehicle treatment (Figure 4b, Supplementary Table S2) and associated with innate immune cell sensing, adhesion and signaling (e.g., *Tlr2, Tlr4, Tlr13, Itgam, Ccl6*, *Ccl9*), consistent with the effects of GW2580 in reducing microglial numbers.

To further probe pathways that may underlie the beneficial effects of GW2580 treatment in an unbiased manner, we undertook gene set enrichment analysis using Camera (Wu & Smyth, 2012). Overall, 949 gene sets were significantly altered following GW2580 treatment (Supplementary Table S2; FDR <0.1; 78 upregulated, 871 downregulated). Of the significantly downregulated gene sets, we found those with the greatest magnitude of change to be involved in immune cell responses and signaling (Figure 4c), including interferon signaling, pattern recognition activity, neuroinflammatory response, phagocytosis and leukocyte activation and migration.

We next wanted to gain further insight to cell specific gene signatures that may be altered and second to this, to verify whether microglia‐related changes are dominant in these tissues. To address this, gene set enrichment analysis was performed on cell type specific gene sets (Table 2; Supplementary Tables S3 and S4). Gene sets for microglia, endothelial cells, oligodendrocytes, oligodendrocyte precursor cells (OPCs) and astrocytes were derived from a published database, comprised of genes with >10‐fold enrichment in expression for a particular cell type, with the exception of oligodendrocyte gene sets which had >3‐fold enrichment (Zhang et al., 2014; Zhang et al., 2016). In support of our findings that microglial alterations are prominent within hypoperfused white matter, we found significant over‐representation of a microglia‐gene set in hypoperfused white matter compared to shams (*n* = 360 genes, FDR = 6.14 × 10^−16^, *P* = 3.32 × 10^−17^). In addition, an endothelial‐associated gene set was found to be significantly over‐represented in hypoperfused white matter compared to shams (*n* = 315 genes, FDR = 2.52 × 10^−6^, *P* = 4.77 × 10^−7^). Astrocyte‐ and OPC‐associated gene sets were also significantly over‐represented but to a lesser extent (astrocyte genes *n* = 71 FDR = 0.0184, *p =* .01; OPC‐associated genes *n* = 12, FDR = 0.009, *p =* .005) while myelinating oligodendrocyte‐associated gene set remained unchanged. Interestingly, following GW2580 treatment, there was significantly reduced representation of a microglial‐related gene set (*n* = 357 genes, FDR 1.50 × 10^−30^, *P* = 4.05 × 10^−32^), and to a lesser extent endothelial‐related gene set (*n* = 315 genes, FDR 8.05 × 10^−4^, *P* = 1.52 × 10^−4^) in hypoperfused white matter. There was also significant over‐representation of a gene set associated with myelinating oligodendrocytes (FDR = 0.058, *p =* .031). Other cell‐associated gene sets related to astrocytes and OPCs were unchanged in hypoperfused white matter with GW2580 treatment. Overall, the data demonstrate inhibition of CSF1R modifies the immune‐related transcriptome profile of hypoperfused white matter particularly of microglia and endothelial genes.

**TABLE 2:** Analysis of cell‐type specific gene sets indicates over‐representation of microglial‐, endothelial‐, OPC‐ and astrocyte‐enriched gene sets following chronic hypoperfusion. Microglial, endothelial and oligodendrocyte gene sets are modified by GW2580 treatment. Cell‐type specific gene sets contain genes that are >10‐fold enriched for a specific cell type with the exception of myelinating oligodendrocyte gene sets, which contain genes that are >3‐fold enriched. Genes included in gene sets are detailed in Supplementary Tables 2 and 3.

Gene set	Sham vs Hypoperfused				Hypoperfused vs Hypoperfused + GW2580			
	Number of genes	Direction	*p* value	FDR	Number of genes	Direction	*p* value	FDR
Microglia	360	Up	3.32 × 10^−17^	6.14 × 10^−16^	357	Down	4.05 × 10^−32^	1.30 × 10^−30^
Endothelial cells	315	Up	4.77× 10^−7^	2.52 × 10^−6^	315	Down	1.52 × 10^−4^	8.05 × 10^−4^
Oligodendrocyte precursor cells	12	Up	0.005	0.009	11	Down	0.085	0.125
Myelinating oligodendrocytes	34	Up	0.854	0.854	34	Up	0.031	0.058
Astrocytes	71	Up	0.0119	0.0184	63	Down	0.04658	0.5069

### 3.5 CSF1R inhibition reduces phagocytic microglia in hypoperfused white matter

**FIGURE 5:**
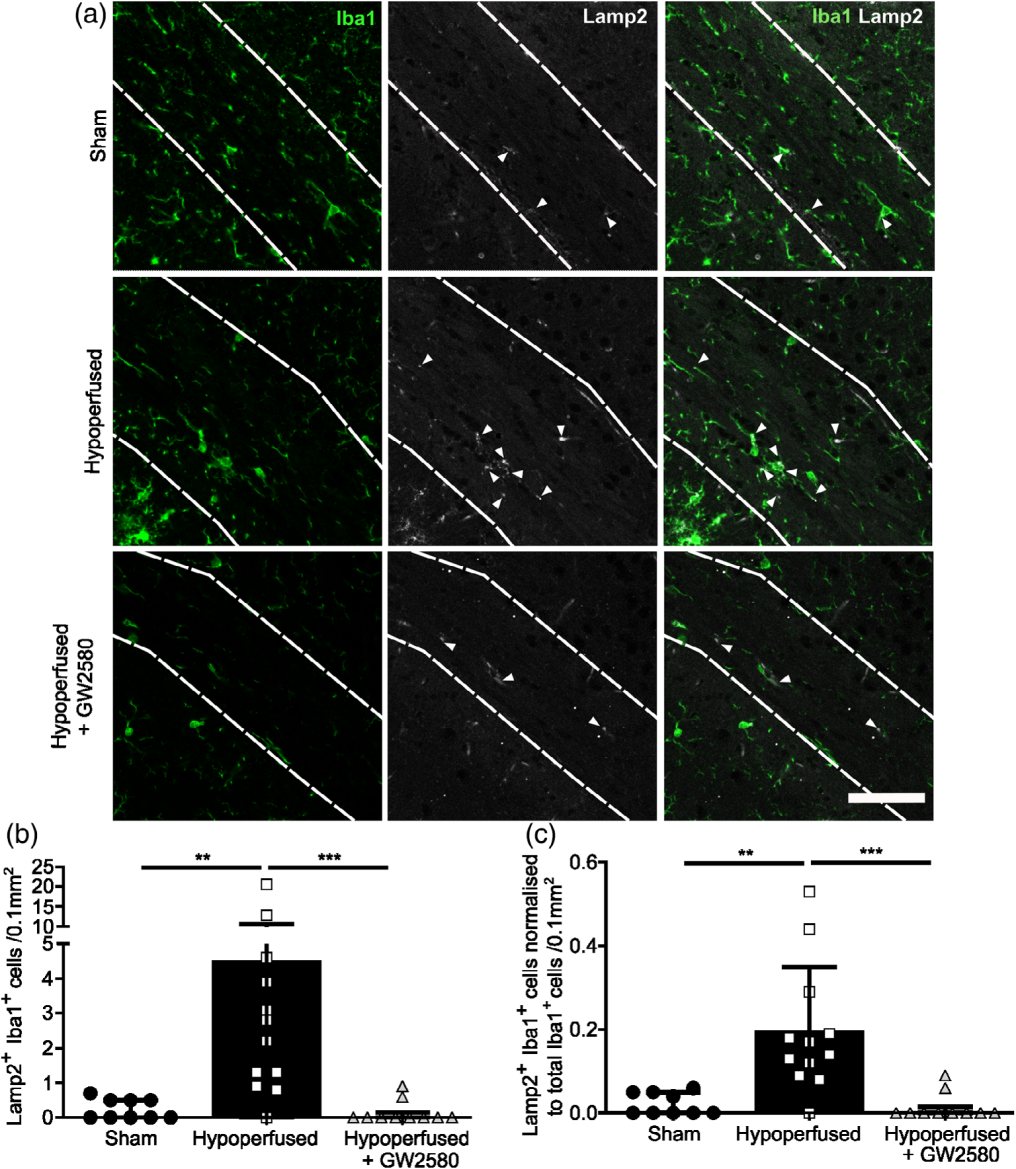
CSF1R inhibition reduces phagocytic microglia in hypoperfused white matter (a) Representative images of Iba1+ /Lamp2+ staining in the corpus callosum following chronic hypoperfusion and GW2580 treatment. Arrowheads indicate Lamp2^+^ positive staining. Scale bar = 50 μm. (b) Quantification of the total number of Lamp2^+^/ Iba1^+^ microglial cells in the corpus callosum of sham (*n* = 9), hypoperfused (*n* = 12) and hypoperfused mice treated with GW2580 (*n* = 10). (c) Normalization of the number of Lamp2^+^ /Iba1^+^ cells to the total number of Iba1^+^ cells in the corpus callosum. Data presented as median ± IQR and analyzed by Kruskal‐Wallis with post hoc Dunn's test. **p < .01, ***p < .001.

Of the immune related gene pathways altered with GW2580 treatment, the impact that treatment had on phagocytosis was of particular interest given the association of aberrant microglial phagocytosis with a number of white matter diseases (Zhang et al., 2020). This phagocytosis gene set was also found to be significantly down‐regulated with GW2580 treatment (FDR = 3.22 × 10^−5^, *P =* 8.70 × 10^−8^). We investigated expression of lysosomal‐associated membrane protein 2 (Lamp2), a lysosomal marker, in microglia following chronic hypoperfusion. There was an overall effect of treatment group (X (2) = 18.307, *p <* .001) on the number of Lamp2‐expressing Iba1^+^ microglia within the corpus callosum. Following chronic hypoperfusion, Lamp2^+^ Iba1^+^ cell numbers increased significantly (4.5 ± 5.8 cells/0.1 mm^2^) compared to sham (0.2 ± 0.3 cells/0.1 mm^2^) (*p <* .01; Figure 5a,b), suggestive of increased phagocytic activity. To confirm that this was not influenced by increased microglial density following chronic hypoperfusion, we normalized the number of Lamp2‐expressing Iba1^+^ cells to the total Iba1^+^ cell number. Once again, we saw an overall effect of treatment (X (2) = 18.27, *p <* .001) with *post hoc* analysis confirming a significant increase in normalized Lamp2^+^ Iba1^+^ density in the corpus callosum (Figure 5c; *p <* .01). GW2580 treatment significantly reduced the number of Lamp2^+^ Iba1^+^ cells in the corpus callosum (0.2 ± 0.3 cells/0.1 mm^2^) (Figure 5b; *p <* 0.001), which was also confirmed following normalization of cell counts to total Iba1^+^ density (Figure 5c; *p <* .001).

### 3.6 CSF1R inhibition ameliorates chronic hypoperfused white matter damage

**FIGURE 6:**
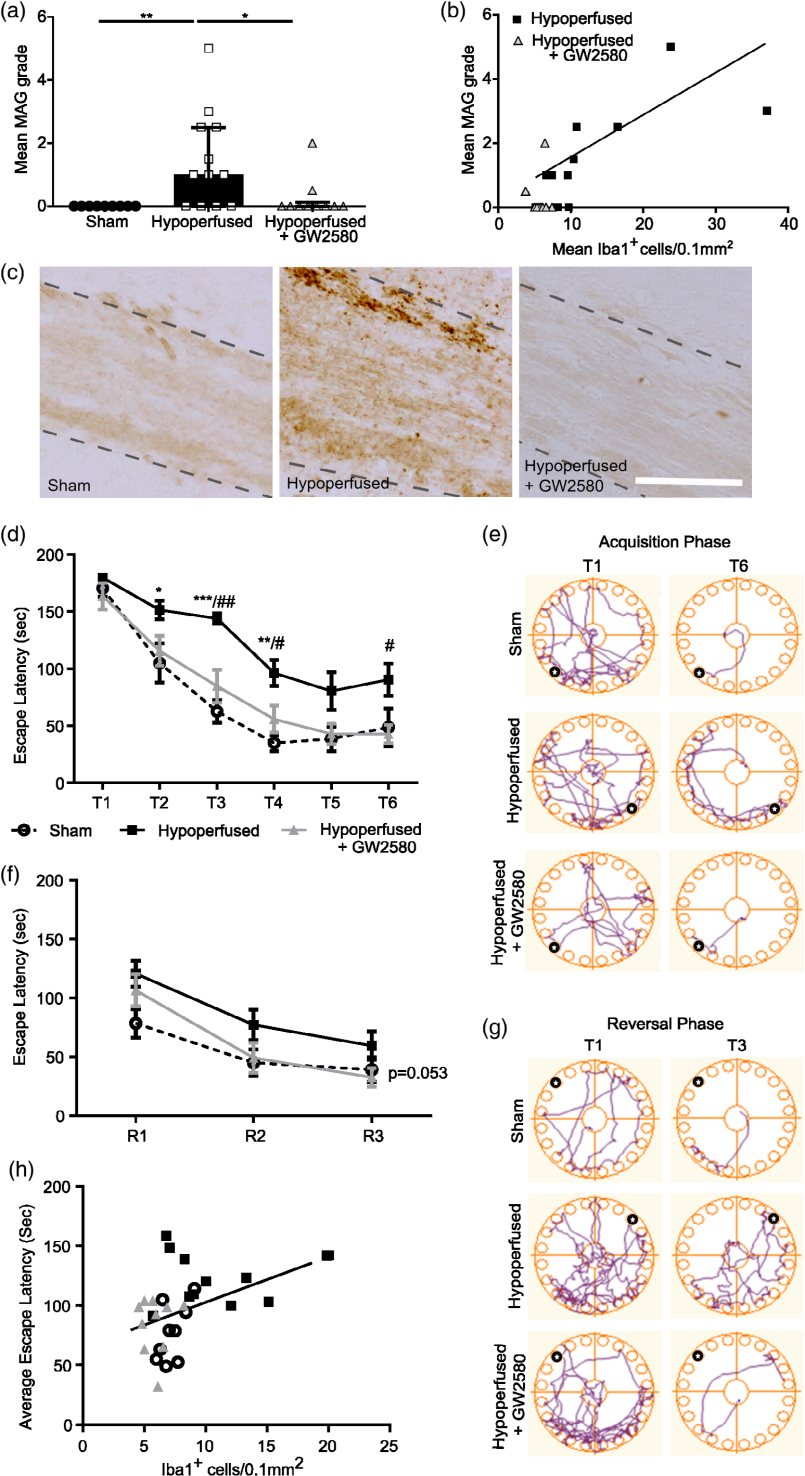
CSF1R inhibition following chronic hypoperfusion improves white matter integrity and cognitive abilities. (a) Semi‐quantitative grading of white matter damage using MAG staining in the corpus callosum following chronic hypoperfusion and GW2580 treatment. Data presented as median ± IQR and analyzed by Kruskal‐Wallis with *post hoc* Dunn's test, *p < .05, **p < .01. (b) White matter damage evidenced by increased MAG grading positively correlates with microglial density post‐hypoperfusion and is rescued by GW2580 treatment. Data analyzed by Spearman's Rho test. (c) Representative images of MAG staining in the corpus callosum. Scale bar = 50 μm. (d) Quantification of latency to escape chamber (seconds) by sham (*n* = 9), hypoperfused (*n* = 12) and hypoperfused mice treated with GW2580 (*n* = 10) across the 6 training days in the acquisition phase of the Barnes maze. Each training day represents an average of two trials, maximum trial length 180 seconds. (e) Representative examples of movement traces across trial days in the acquisition phase. Black circle indicates location of escape hole. (f) Quantification of latency to escape chamber (seconds) across the 3 training days in the reversal phase of the Barnes maze. (g) Representative examples of movement traces across trial days in the reversal phase. Black circle indicates location of escape hole. Data (d, f) presented as mean ± SEM and analyzed by repeated measures two‐way ANOVA with post hoc Bonferroni correction *p < .05, **p < .01, ***p < .001, ^#^ *p* < .05, ^##^ *p* < .01. * sham versus. hypoperfused, # hypoperfused versus hypoperfused + GW2580. (h) Impaired spatial learning evidenced by increased latency to escape chamber following chronic hypoperfusion correlates positively with increased microglial density in the corpus callosum. Data analyzed by Spearman's Rho.

We next aimed to determine whether microglial proliferation via CSF1R activation post‐hypoperfusion plays a causal role in white matter disease and whether the effective blockade of this could rescue hypoperfusion‐induced white matter damage. White matter damage was assessed by the extent of myelin associated glycoprotein (MAG) immunohistochemistry in the corpus callosum. Overall, we found a significant effect of treatment (X (2) = 11.65, *p <* .01). White matter damage was significantly increased post‐hypoperfusion (Figure 6a,c; *p <* .01), but notably was reduced with GW2580 treatment (*p <* .05). Furthermore, the extent of white matter disruption was significantly related to microglial number (Figure 6b; Spearman *r* = 0.644, *p <* .01) with low numbers of microglia (as seen in sham and GW2580‐treated hypoperfused mice) linked with less extensive white matter damage. Collectively, these data illustrate that hypoperfusion‐induced white matter disruption is coupled to microglial population expansion and such disruption can be prevented through CSF1R inhibition.

### 3.7 CSF1R inhibition rescues spatial learning impairments in hypoperfused mice

White matter disruption has been shown clinically to be most closely associated with cognitive impairment (Prins & Scheltens, 2015). In preclinical models, our group and others have demonstrated that hypoperfusion‐induced white matter impairments cause spatial learning and memory deficits (Coltman et al., 2011; Holland et al., 2015; Kitamura et al., 2017; Shibata et al., 2007). Since GW2580 effectively restored white matter integrity through blockade of microglial expansion, we next aimed to determine whether GW2580 treatment post‐hypoperfusion would alleviate cognitive deficits. Spatial learning and cognitive flexibility were assessed using a modified Barnes maze paradigm starting 4 weeks post‐surgery. Spatial learning was measured as the time taken for animals to locate the correct escape hole over six trial days (escape latency; Figure 6d,e). We found a significant effect of time (F_(3.34,93.51)_ = 65.08, *p <* .0001), indicative of spatial learning across trials, and treatment group (F_(2,28)_ = 13.12, *p <* .0001). Although both sham and hypoperfused mice started with similar escape latencies in the initial acquisition trial (T1), vehicle‐treated hypoperfused mice demonstrated significantly longer escape latencies across subsequent trials compared to shams (Figure 6d,e; T2 *p <* .05, T3 *p <* .0001, T4 *p <* .001), indicating impaired spatial learning. GW2580‐treated hypoperfused mice displayed similar escape latencies to shams (Figure 6d,e). Across trials, these were significantly reduced compared to vehicle‐treated hypoperfused mice (Figure 6d,e; T3 *p <* .001, T4 *p <* .05, T6 *p <* .05).

As an additional measure of spatial learning, total distance traveled during each trial was calculated (Supplementary Figure S5b). Reduced travel distance over subsequent trials would be indicative of spatial learning as the mouse would be taking a more direct route to the escape chamber as they learn the location. A significant overall effect of time was identified (F_(5,140)_ = 31.97, *p* < .001), as well as an overall significant group difference (F_(2,28)_ = 7.48, *p* < .01) with no interaction (*p* = .49). *Post hoc* analysis revealed a significant increase in distance traveled, and therefore impaired spatial learning, within vehicle hypoperfused mice in comparison to shams across several trials (T2; *p* < .05, T3; *p* < .01, T4; *p* < .05) (Supplementary Figure S5b). Notably, distance traveled within the hypoperfused GW2580 treated mice was comparable to shams and significantly reduced in comparison to hypoperfusion alone (T2; *p* < .05) (Supplementary Figure S5b). Due to the motile nature of the task, average speed measurements were gathered to ensure that mobility differences did not exist between groups. Overall a significant main effect of time was observed (F_(3.18,88.94)_ = 11.32, *p* < .001). However, no significant effect of group was observed (*p* = .32) as well as no significant interactions (*p* = .084) indicating that escape latency can be used as a measure of learning without speed as a confounding factor (Supplementary Figure S5a).

Further to this, a significant correlation between escape latency and microglial numbers in the corpus callosum was determined (Figure 6h; Spearman *r* = 0.57, *p <* .001). Together these data suggest that inhibition of microglial proliferation using GW2580 rescues hypoperfusion‐induced deficits in spatial learning.

To test cognitive flexibility, mice underwent reversal training in which the location of the escape hole was rotated 180° to the opposite side of the maze. Overall there was a significant effect of time (F_(2,56)_ = 31.98, *p <* .0001), indicating learning across trial days. Although there was a trend for treatment across groups, this failed to reach statistical significance (Figure 6f,g; F_(2,28)_ = 3.28, *p =* .053) but it was notable that both sham and GW2580‐treated hypoperfused mice show reduced escape latencies compared to vehicle‐treated hypoperfused mice. These data suggest that GW2580 treatment may also help to improve cognitive flexibility following hypoperfusion.

Distance traveled was also used as a measure of learning, as with escape latency a significant overall effect of time was observed (F_(2,56)_ = 11.32, *p* < .001), with once again an overall group effect narrowly missing statistical significance (*p* = .053) (Supplementary Figure S5d). Speed was measured throughout the trials to determine if motility could influence the results. Overall, no significant effect of time (*p* = .06) or group was observed (*p* = .12), indicating that motility was comparable across groups (Supplementary Figure S5c).

Collectively we demonstrate that inhibition of CSF1R (by GW2580) reduces microglial proliferation and exerts protective effects on white matter integrity and subsequently cognitive ability, particularly spatial learning, following chronic hypoperfusion.

## 4 DISCUSSION

Microvascular inflammation and increased numbers of activated microglia are strongly linked to white matter abnormalities (Al‐Mashhadi et al., 2015; Hase et al., 2018; Simpson et al., 2007; Simpson et al., 2007; Simpson et al., 2009; Waller et al., 2020; Wardlaw et al., 2019; Wharton et al., 2015). The present study builds on this work to show increased expression of *CSF1R/Csf1r* and genes related to microglia reactivity in abnormal white matter related to cerebrovascular disease both in human and in a mouse model. To address the functional role of the CSF1R pathway and microglial proliferation, we used a mouse model of cerebrovascular disease involving chronic cerebral hypoperfusion (Coltman et al., 2011; Fowler et al., 2018; Holland et al., 2011; Kitamura et al., 2017; Manso et al., 2018; Reimer et al., 2011; Shibata et al., 2004). We, and others, have also previously shown that chronic hypoperfusion is associated with an increase in microglial density in damaged white matter tracts (Ben‐Ari et al., 2019; Coltman et al., 2011; Fowler et al., 2018; Holland et al., 2011; Kitamura et al., 2017; Manso et al., 2018; Miyanohara et al., 2018; Reimer et al., 2011; Zhang et al., 2020). This study provides further confirmation that microglial proliferation is induced with hypoperfusion and sustained over time. Additionally, we show that resident microglia, rather than infiltrating peripheral cells, contribute to the expanded cell population in response to hypoperfusion in white matter, in accordance with our previous data (Manso et al., 2018). The lack of changes in microglia detected in hypoperfused cortical gray matter is also consistent with the comparably reduced sensitivity to hypoxic conditions of gray compared to white matter, including microglia themselves (Hart et al., 2012; Pantoni et al., 1996). There is a growing appreciation that microglial homeostasis and function may be differentially regulated within white and gray matter, from regional sensitivity to CSF1R ligands (Easley‐Neal et al., 2019). identification of white matter‐specific microglial populations (Hagemeyer et al., 2017; Hammond et al., 2018; Li et al., 2018; Wlodarczyk et al., 2017) and their responses to white matter pathology (Lee et al., 2019).

In conjunction with our previous studies showing that microglial density closely correlates with impaired white matter structure and function following hypoperfusion (Fowler et al., 2018; Kitamura et al., 2017; Manso et al., 2018), we report that increased microglial density positively correlates with progressive white matter disruption. These data suggest that proliferation and maladaptive activation of white matter microglia may contribute to disruption of white matter following chronic hypoperfusion. Furthermore, while we and others have shown the beneficial effects of broadly modulating the inflammatory response on white matter integrity and cognition following cerebral hypoperfusion (Fowler et al., 2018; Manso et al., 2018; Miyanohara et al., 2018), to date there have been no studies directly modulating CSF1R. Inhibition of CSF1R using GW2580 restores microglial numbers to sham levels, demonstrating a robust blockade of the proliferative response post‐hypoperfusion. Inhibition of CSF1R signaling restored white matter integrity to sham levels, providing evidence of a direct contribution of microglia to white matter disruption following chronic cerebral hypoperfusion. Other studies have similarly reported effects of GW2580 treatment to reduce microglial density in chronic disease models (Gerber et al., 2018; Gomez‐Nicola et al., 2013; Martinez‐Muriana et al., 2016;Neal et al., 2020; Olmos‐Alonso et al., 2016) with some studies reporting a protective effect in models with white matter damage (Crespo et al., 2011; Garcia‐Agudo et al., 2019; Gerber et al., 2018; Janova et al., 2018; Klein et al., 2015), including that observed in chronic stroke (Jackson et al., 2020). *CSF1R/Csf1r* is expressed by cells of the myeloid lineage and evidence for functional expression of *CSF1R/Csf1r* on non‐myeloid cells is not compelling (Hume et al., 2020). We conducted an assessment of myeloid subsets by FACS which indicated there were no significant differences in the number of inflammatory monocytes, monocyte‐derived cells, or neutrophils in the corpus callosum of hypoperfused mice compared to shams. In contrast there was a prominent increase in microglia. Thus, our data suggest a largely microglial‐restricted expansion post‐hypoperfusion would be the key target of CSF1R inhibition. However, we cannot discount that there may be effects on peripheral myeloid populations that could impact on disease progression. Further we do not exclude possible effects of inhibiting CSF1R on microglial function independently of blocking proliferation (Perez et al., 2023; Soto‐Diaz et al., 2021), which may include altering their sensitivity to activation by other stimuli and chemotactic function.

A number of studies, including our own, have shown cerebral hypoperfusion culminates in cognitive impairments particularly spatial learning and memory (Ben‐Ari et al., 2019; Coltman et al., 2011; Holland et al., 2015; Nishio et al., 2010; Shibata et al., 2007). The Barnes maze is a well characterized method to assess spatial learning acquisition and cognitive flexibility. We (Li et al., 2021) and others have reported the sensitivity of this method to assess spatial learning acquisition and cognitive flexibility in the hypoperfusion model and in other models in which white matter is compromised (McNamara et al., 2023). Consistent with these studies we show impaired spatial learning acquisition and cognitive flexibility, whilst motor abilities were not impaired. Importantly our data show a robust rescue of spatial learning following GW2580 treatment to performance levels similar to sham controls. Cognitive flexibility was modestly improved by GW2580 and likely reflects the more challenging requirement of mice to re‐learn a new location. There is a close relationship between white matter damage and cognitive abilities post‐hypoperfusion (Ben‐Ari et al., 2019; Coltman et al., 2011; Holland et al., 2015; Nishio et al., 2010; Shibata et al., 2007), and while some studies have reported the beneficial impact of anti‐inflammatory intervention (Miyanohara et al., 2018; Qin et al., 2017), the relationship between microglial density and impaired cognition has not yet been reported. Here, we show that increased microglial density closely correlates with impairments in spatial learning. The observed protection likely results from preservation of white matter structure. Spatial learning acquisition and cognitive flexibility are dependent on the integrity of frontal cortical circuitry (Coltman et al., 2011; Shibata et al., 2007). Depletion of microglia prior to the onset of hypoperfusion using the CSF1R inhibitor PLX3397 reduces white matter damage and associated cognitive impairment (Kakae et al., 2019). However, depletion of microglia is not always beneficial as shown by a study in which mice were treated with the CSF1R inhibitor PLX5622 which abolished microglia and the protective effects of ischaemic preconditioning in white matter (Hamner et al., 2022). In this study, we specifically selected the dose of GW2580 as it does not ablate microglia (Neal et al., 2020; Olmos‐Alonso et al., 2016). This approach, inhibition of CSF1R with GW2580, prevents microglial proliferation without affecting their survival (Neal et al., 2020; Olmos‐Alonso et al., 2016), and is more translatable to a clinical setting given that complete elimination of microglia is unlikely to be desirable. Increased microglial numbers are also observed in the chronic post‐stroke response in white matter and in this condition, depletion of microglia by shRNA targeting of *Csf1r* has protective effects on white matter integrity and cognitive abilities (Jackson et al., 2020). Collectively, the data suggests sustained microglial proliferation mediates white matter damage and contributes to vascular cognitive impairment.

It is not fully understood how microglia contribute to white matter damage post‐hypoperfusion. Our data indicate that hypoperfusion rapidly induces microglial proliferation, likely in response to hypoxic conditions in the white matter (Duncombe et al., 2017; Manso et al., 2018; Reimer et al., 2011). What remains unclear is the mechanism by which microglia cause white matter damage. To address this, we undertook a bulk transcriptomic analysis of white matter. Investigation of the most altered genes between sham and hypoperfused white matter indicated that the majority were increased and overlapped with genes we had previously identified as altered in a microarray study of white matter 72 hour post‐hypoperfusion (Reimer et al., 2011), including many related to microvascular inflammation (e.g., *Il4ra*, *Angpt2*, *vWF*, *Osmr*, and *Dll4)*. The clinical relevance of these changes is highlighted by the presence of similar molecular and cellular alterations in white matter of the aging brain (Simpson et al., 2007; Simpson et al., 2007; Simpson et al., 2009). A recent GWAS of white matter biomarkers in cerebrovascular disease has also revealed alterations in immune cell genes including human leukocyte antigen B (*HLA‐B*), major histocompatibility complex I (*MHC I* and *HLA‐S*), cell‐surface proteins involved in immune system regulation (Persyn et al., 2020). Alongside this, a transcriptome‐wide association study (TWAS) identified 66 genes associated with the same neuroimaging parameters, with 10 of these being highly enriched in immune cells, further supporting a key role for inflammation in cerebrovascular disease pathogenesis (Barbu et al., 2020; Persyn et al., 2020). Interestingly, following stratification of WMHs, risk loci in many of these immune‐related genes are associated only with periventricular WMHs and not deep WMHs, including *NMT1, NEURL1*, and *UNC13D* (Armstrong et al., 2020), which are highly enriched in CNS microglia/macrophages (Zhang et al., 2014). Consistent with the effects of GW2580 treatment, the majority of genes, associated with inflammatory processes and signaling (e.g., *Tlr2, Tlr4, Tlr13, Itgam, Ccl6*, *Ccl9*), were reduced compared to vehicle treatment post‐hypoperfusion.

In addition, a key outcome of our RNAseq analysis was identifying predominantly microglial, but also endothelial‐enriched, gene sets to be significantly increased in response to hypoperfusion and subsequently modified by GW2580 treatment. While we saw minor changes in OPC and astrocytic‐enriched gene sets post‐hypoperfusion, these were unaltered following GW2580 treatment. Our immunohistochemical data also supported modest changes in astrogliosis. Interestingly, oligodendrocyte‐enriched gene sets were mostly unchanged following hypoperfusion but were significantly increased following GW2580 treatment. These data suggest that, of the components of the glial‐vascular unit, microglia and endothelial cells are most affected by hypoperfusion, and thus key candidates to influence downstream pathology. Of interest was the identification of endothelial‐enriched gene sets in response to hypoperfusion and thereafter their modification by GW2580. Increasing preclinical and clinical evidence implicates endothelial dysfunction as an initiator of cerebrovascular disease (Iadecola et al., 2019; Rajani & Williams, 2017; Wang et al., 2018; Wardlaw et al., 2019) and early changes in endothelial gene expression have been reported in the BCAS model (Duncombe et al., 2017). Interestingly, the present study suggests that an intervention targeting CSF1R, a myeloid cell‐restricted receptor, has an effect on endothelial cells and suggests microglia can regulate endothelial cell responses.

Interrogation of the pathways that are altered with GW2580 highlighted that significantly downregulated pathways with the greatest magnitude of change were involved in the inflammatory response, including interferon signaling, pattern recognition activity, neuroinflammatory response, phagocytosis, and leukocyte activation/migration. We were particularly interested in following up evidence of phagocytosis in white matter since aberrant phagocytosis by microglia has been linked to white matter disease (Zhang et al., 2020). In particular, a study elegantly demonstrated, using CLARITY imaging, that increased numbers of activated and phagocytic microglia (CD86^+^/CD68^+^) directly contact and phagocytose intact myelin fibers, potentiating white matter damage after chronic cerebral hypoperfusion in a rat model (Zhang et al., 2020). Subsequent analysis revealed that the complement C3‐C3aR pathway was important in mediating this aberrant microglial activity. Human deep subcortical white matter lesions are also characterized by the presence of CD68^+^ microglia (Waller et al., 2019), suggestive of increased phagocytic activity. It has been shown that phagocytosis of myelin or myelin debris is also sufficient to induce microglial dysfunction (Safaiyan et al., 2016). In keeping with this, our data show an increased density of Lamp2^+^ microglia and widespread white matter damage following chronic hypoperfusion. Thus, we suggest that in our study increased microglial activity and phagocytosis could contribute to myelin damage, trigger further microglial dysfunction and result in propagation of white matter damage. The near absence of white matter damage following GW2580 treatment supports this hypothesis. Our RNAseq data indicated GW2580 treatment decreased representation of phagocytosis‐associated genes. Although changes in gene expression in bulk RNAseq could be influenced by microglial density following CSF1R inhibition, the quantitative cellular changes in Lamp2^+^/Iba1^+^ counts provide direct evidence that GW2580 reduces a phagocytic activity marker. Follow up studies could determine whether inhibition of microglia proliferation specifically affects their phagocytic function.

There are a few confounds and/or limitations of this study to note. At the outset of the study a number of vehicle treated hypoperfused mice were excluded due to poor recovery post‐surgery. There were no GW2580 treated mice that were excluded suggesting GW2580 may improve survival post‐hypoperfusion. Since we studied survivors post‐surgery and a number of vehicle hypoperfused mice were not able to be studied on outcome measures we are also likely to be underestimating the true effect of GW2580 hypoperfusion. The study was restricted to one time point and to increase the translational relevance of the study it would be important to assess the effect of GW2580 longitudinally to assess longer term consequences and in addition with comorbidities. Similarly, more extensive behavioral testing using a repertoire of tasks should be used to assess the broader impact of GW2580 treatment on various aspects of cognition. Further the present study focused on bulk RNA sequencing of white matter tissues to provide a global overview of alterations in gene expression. However more sophisticated single cell RNA sequencing analysis could provide spatiotemporal insight to how hypoperfusion influences cell heterogeneity and how these may change with disease progression and in response to CSF1R inhibition. A single cell resolution approach would be particularly relevant as our data point to the presence/emergence of discrete subsets of microglia following hypoperfusion.

In conclusion, we have demonstrated that expression of *CSF1R/Csf1r* and indices of microglial reactivity are increased and related to the extent of white matter pathology in human and mouse cerebrovascular disease. Second to this, we show that targeting microglial proliferation, via CSF1R inhibition, effectively rescues white matter integrity and restores cognitive function associated with modulation of immune cell pathways, including phagocytosis. We suggest that microglial proliferation and downstream inflammatory related pathways play a major causative role in vascular white matter disease and propose inhibition of CSF1R as a target for the treatment of chronic cerebrovascular disease.

## AUTHOR CONTRIBUTIONS

Katharine E. Askew, Joshua Beverley, Emma Sigfridsson, Jessica Duncombe, Edel Hennessy, Stefan Szymkowiak, Juraj Koudelka inputted to the experimental design overseen by Karen Horsburgh. Karen Horsburgh performed the surgery supported by Jessica Duncombe who performed the laser speckle imaging and analysis. Joshua Beverley, Emma Sigfridsson conducted the behavioral studies and the analysis. Katharine E. Askew was responsible for FACS, qPCR and analysis supported by Stefan Szymkowiak. Joshua Beverley, Emma Sigfridsson, Jessica Duncombe, Edel Hennessy, Juraj Koudelka were responsible for the pathology studies and analysis. Katherine Emelianova, Owen Dando, performed the processing and analysis of the bulk sequencing data overseen by Giles E. Hardingham. Neshika Samarasekera, Rustam Al‐Shahi Salman, Colin Smith provided the human samples and clinical data. Diego Gomez‐Nicola contributed expertise on use of GW2580 in mouse model and interpretation of data. Adriana A. S. Tavares, Raj N. Kalaria, Barry W. McColl and Karen Horsburgh obtained the funding for this project and overseen the studies within the manuscript. Katharine E. Askew and Karen Horsburgh wrote the initial draft of the manuscript to which all authors contributed.

## FUNDING INFORMATION

We gratefully acknowledge grant support from Alzheimer's Research UK (ARUK) (ARUK‐PG2016B‐6), Alzheimer's Society (290 [AS‐PG‐15b‐018]; 228 [AS‐DTC‐2014‐017]), Medical Research Council UK (MR/L003384/1, MR/R001316/1) and the UK Dementia Research Institute (UK DRI). UK DRI receives its funding from the Medical Research Council, Alzheimer's Society, and Alzheimer's Research UK The LINCHPIN study (Lothian IntraCerebral Hemorrhage, Pathology, Imaging and Neurological Outcome) was funded by UK Medical Research Council and The Stroke Association. Joshua Beverley and Emma Sigfridsson were supported by Alzheimer's Society Doctoral Training PhD studentships and the RS McDonald Charitable Trust.

## CONFLICT OF INTEREST STATEMENT

The authors have declared that no competing interests exist.

## Supporting information


**Supplementary Figure S1:** Cortical cerebral blood flow is reduced post‐BCAS. (a) BCAS surgery reduced CBF compared to sham at 24 hours and 6 days. (b) Representative images of laser speckle flowmetry in sham and BCAS at baseline, 24 hours and 6 days. (c) BCAS surgery reduced CBF compared to sham at 24 hours and 6 weeks and to a similar extent in the GW2580 treated group. (d) Representative images of laser speckle flowmetry in sham, BCAS vehicle and BCAS GW2580 animals at baseline, 24 hours and 6 weeks. Mean ± SEM. ***p < .001 (* indicates *post hoc* differences between sham and BCAS vehicle), ^###^ *p* < .001 (^#^ indicates *post hoc* differences between sham and BCAS GW2580).
**Supplementary Figure S2:** (a) Full gating strategy and representative flow cytometry dot plots identifying neutrophil (Ly6G^+^), monocyte (Ly6C^+^), microglia (CD11b^+^ CD45^low^ Ly6C^−^ Ly6G^−^) and macrophage (CD11b^+^ CD45^high^ Ly6C^−^ Ly6G^−^) populations 7 days post‐surgery. (b) Flow cytometric quantification of the absolute numbers of microglia, macrophages, neutrophils and monocytes in the gray matter of sham (*n* = 3) and hypoperfused (*n* = 6) mice, based on the gating strategy shown in (a). There are no significant differences in these numbers between sham and hypoperfused mice.
**Supplementary Figure S3:** CSF1R inhibition following chronic hypoperfusion prevents expansion of microglia in white matter regions. (a and b) Quantification of the number of microglial cells (Iba1^+^) in the internal capsule (a) and fimbria (b); (c and d) Iba1% area staining as a measure of microglial activation in the internal capsule (c) and fimbria (d) following 6 weeks of hypoperfusion and GW2580 treatment. (e) Quantification of the number of proliferating microglial cells (Iba1^+^ Ki67^+^) in the fimbria following chronic hypoperfusion and GW2580 treatment. Data presented as mean ± SD and analyzed by one‐way ANOVA with *post hoc* Bonferroni correction, *p < .05, **p < .01.
**Supplementary Figure S4:** CSF1R inhibition following chronic hypoperfusion modestly reduces astrogliosis in white matter regions. (a) Astrogliosis was increased in the hypoperfused vehicle group compared to shams and the hypoperfused GW2580 group in the corpus callosum. Astrogliosis was not significantly altered in the fimbria (b) and the internal capsule (c). Data presented as mean ± SD and analyzed by one‐way ANOVA with *post hoc* Bonferroni correction, *p < .05.
**Supplementary Figure S5:** Movement speed is unaffected by hypoperfusion or GW2580 treatment. (a) Quantification of movement speed (metres per second) across the 6 training days in the acquisition phase of the Barnes maze. Each training day represents an average of 2 trials. (b) Quantification of total distance traveled (m) across the 6 training days in the acquisition phase. (c) Quantification of movement speed (metres per second) across the 3 training days in the reversal phase of the Barnes maze. Each training day represents an average of 2 trials. (d) Quantification of total distance traveled (m) across the 3 training days in the reversal phase. Data presented as mean ± SEM and analyzed by repeated measures two‐way ANOVA with *post hoc* Bonferroni correction. *p < .05, **p < .01, ^#^ *p* < .05, * sham versus hypoperfused, # hypoperfused versus hypoperfused + GW2580.

**Table S1:** Supplementary Table 1.

**Table S2:** Supplementary Table 2.

**Table S3:** Supplementary Table 3.

**Table S4:** Supplementary Table 4.

## Data Availability

Data supporting the findings of this study are available within the article or Supplementary material. The data are available from the corresponding author upon reasonable request.
