# Supplementary material for: Inhibiting CSF1R alleviates cerebrovascular white matter disease and cognitive impairment
Source: Glia. 2023 Nov 1;72(2):375–95. doi: 10.1002/glia.24481 (PMC10952452; doi:10.1002/glia.24481)
Supplement: Supplementary file 1 — Supplementary Figure S1: Cortical cerebral blood flow is reduced post‐BCAS. (a) BCAS surgery reduced CBF compared to sham at 24 hours and 6 days. (b) Representative images of laser speckle flowmetry in sham and BCAS at baseline, 24 hours and 6 days. (c) BCAS surgery reduced CBF compared to sham at 24 hours and 6 weeks and to a similar extent in the GW2580 treated group. (d) Representative images of laser speckle flowmetry in sham, BCAS vehicle and BCAS GW2580 animals at baseline, 24 hours and 6 weeks. Mean ± SEM. ***p < .001 (* indicates post hoc differences between sham and BCAS vehicle), ### p < .001 (# indicates post hoc differences between sham and BCAS GW2580). Supplementary Figure S2: (a) Full gating strategy and representative flow cytometry dot plots identifying neutrophil (Ly6G+), monocyte (Ly6C+), microglia (CD11b+ CD45low Ly6C− Ly6G−) and macrophage (CD11b+ CD45high Ly6C− Ly6G−) populations 7 days post‐surgery. (b) Flow cytometric quantification of the absolute numbers of microglia, macrophages, neutrophils and monocytes in the gray matter of sham (n = 3) and hypoperfused (n = 6) mice, based on the gating strategy shown in (a). There are no significant differences in these numbers between sham and hypoperfused mice. Supplementary Figure S3: CSF1R inhibition following chronic hypoperfusion prevents expansion of microglia in white matter regions. (a and b) Quantification of the number of microglial cells (Iba1+) in the internal capsule (a) and fimbria (b); (c and d) Iba1% area staining as a measure of microglial activation in the internal capsule (c) and fimbria (d) following 6 weeks of hypoperfusion and GW2580 treatment. (e) Quantification of the number of proliferating microglial cells (Iba1+ Ki67+) in the fimbria following chronic hypoperfusion and GW2580 treatment. Data presented as mean ± SD and analyzed by one‐way ANOVA with post hoc Bonferroni correction, *p < .05, **p < .01. Supplementary Figure S4: CSF1R inhibition following chronic hypoperfusion m [file GLIA-72-375-s002.docx]

**
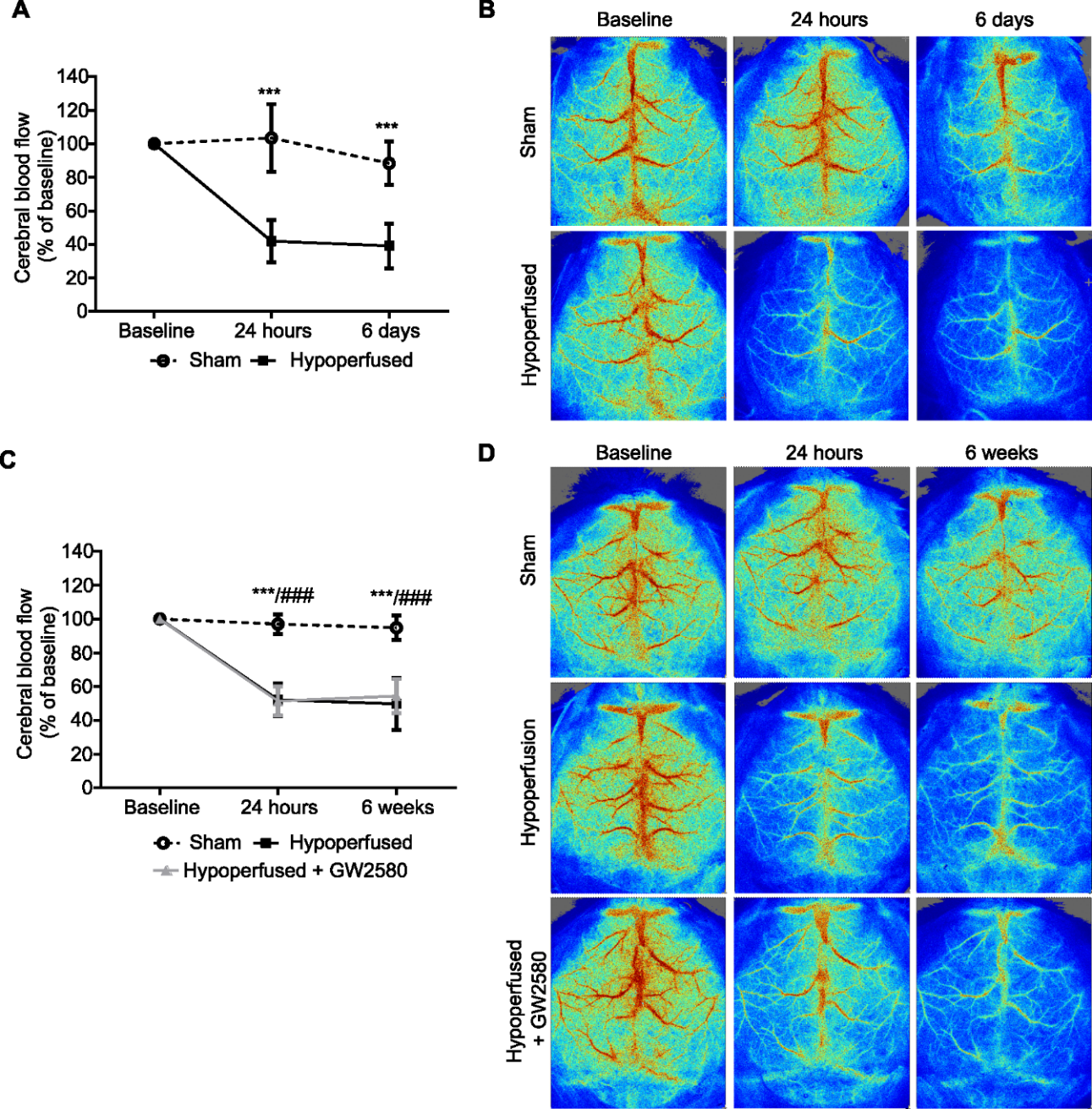
Supplementary Figures**

**Supplementary Figure 1. Cortical cerebral blood flow is reduced post-BCAS. (A)** BCAS surgery reduced CBF compared to sham at 24hr and 6 days. **(B)** Representative images of laser speckle flowmetry in sham and BCAS at baseline, 24 hours and 6 days.(**C**) BCAS surgery reduced CBF compared to sham at 24hr and 6 weeks and to a similar extent in the GW2580 treated group. **(D)** Representative images of laser speckle flowmetry in sham, BCAS vehicle and BCAS GW2580 animals at baseline, 24 hours and 6 weeks. Mean±SEM. ***p<0.001 (* indicates *post hoc* differences between sham and BCAS vehicle), ^###^p<0.001 (^#^ indicates *post hoc* differences between sham and BCAS GW2580).

**
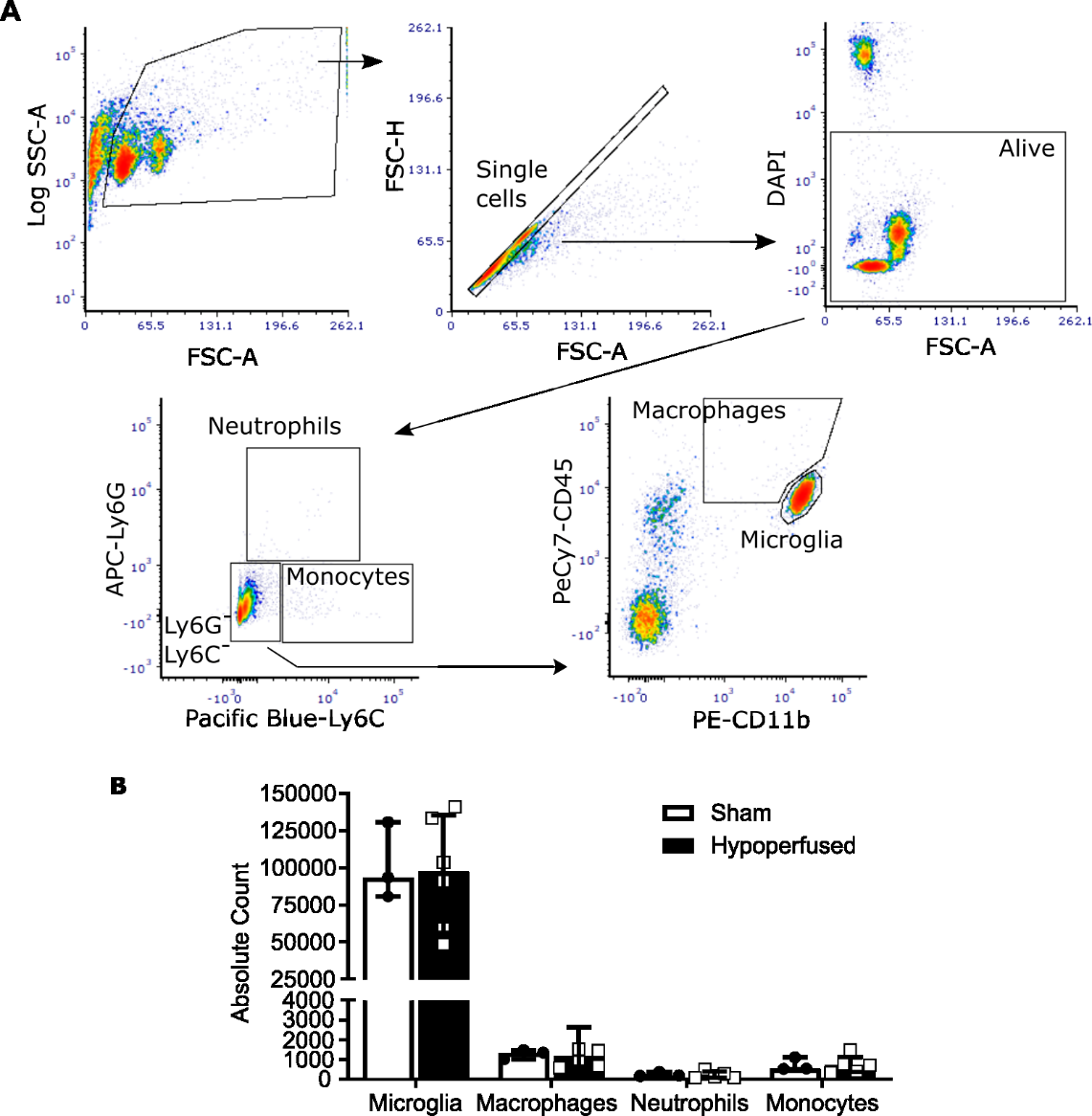
**

**Supplementary Figure 2. (A)** Full gating strategy and representative flow cytometry dot plots identifying neutrophil (Ly6G^+^), monocyte (Ly6C^+^), microglia (CD11b^+^ CD45^low^ Ly6C^-^ Ly6G) and macrophage (CD11b^+^ CD45^high^ Ly6C^-^ Ly6G^-^) populations 7 days post-surgery. **(B)** Flow cytometric quantification of the absolute numbers of microglia, macrophages, neutrophils and monocytes in the grey matter of sham (n=3) and hypoperfused (n=6) mice, based on the gating strategy shown in **A**. There is no significant differences in these numbers between sham and hypoperfused mice.


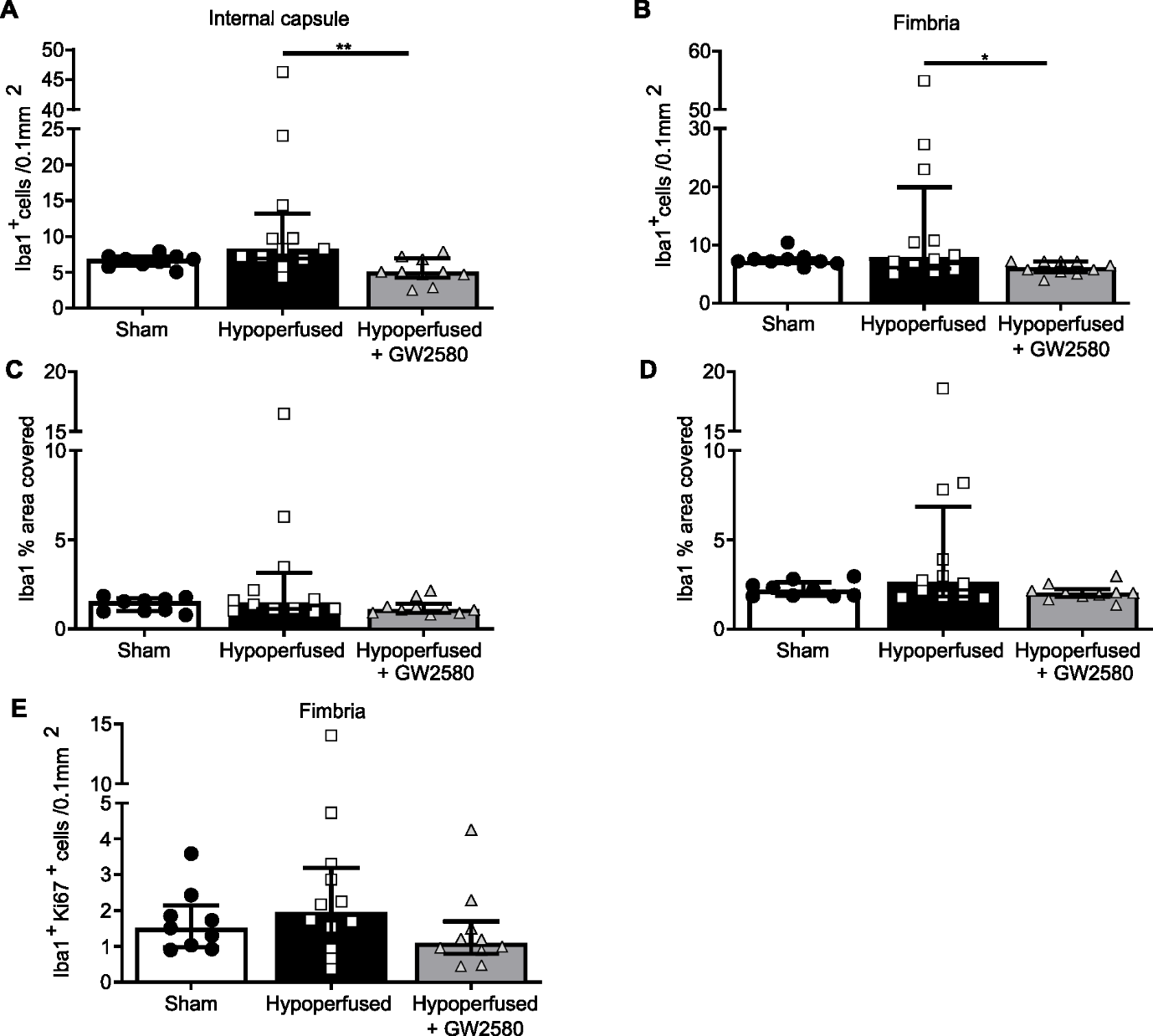


**Supplementary Figure 3 CSF1R inhibition following chronic hypoperfusion prevents expansion of microglia in white matter regions. (A,B)** Quantification of the number of microglial cells (Iba1^+^) in the internal capsule (**A**) and fimbria (**B**); **(C,D)** Iba1% area staining as a measure of microglial activation in the in the internal capsule (**C**) and fimbria (**D**) following 6 weeks of hypoperfusion and GW2580 treatment. **(E)** Quantification of the number of proliferating microglial cells (Iba1^+^ Ki67^+^) in the fimbria following chronic hypoperfusion and GW2580 treatment. Data presented as mean ± SD and analysed by one-way ANOVA with *post hoc* Bonferroni correction, *p<0.05, **p<0.01.


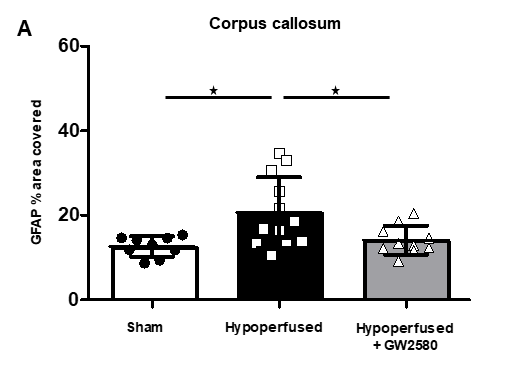


**Fimbria**

**B**

**Internal capsule**

**C**

**Supplementary Figure 4 CSF1R inhibition following chronic hypoperfusion modestly reduces astrogliosis in white matter regions. (A)** Astrogliosis was increased in the hypoperfused vehicle group compared to shams and the hypoperfused GW2580 group in the corpus callosum. Astrogliosis was not significantly altered in the fimbria **(B)** and the internal capsule **(C)**. Data presented as mean ± SD and analysed by one-way ANOVA with *post hoc* Bonferroni correction, *p<0.05.

**
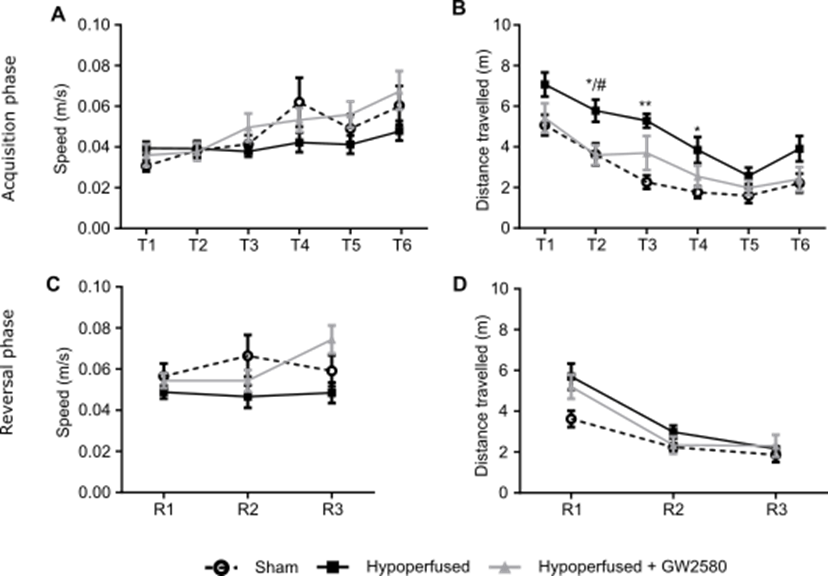
**

**Supplementary Figure 5: Movement speed is unaffected by hypoperfusion or GW2580 treatment. (A)** Quantification of movement speed (metres per second) across the 6 training days in the acquisition phase of the Barnes maze. Each training day represents an average of 2 trials. **(B)** Quantification of total distance travelled (m) across the 6 training days in the acquisition phase. **(C)** Quantification of movement speed (metres per second) across the 3 training days in the reversal phase of the Barnes maze. Each training day represents an average of 2 trials. **(D)** Quantification of total distance travelled (m) across the 3 training days in the reversal phase. Data presented as mean ± SEM and analysed by repeated measures two-way ANOVA with *post hoc* Bonferroni correction. *p<0.05, **p<0.01, ^#^p<0.05, * sham vs. hypoperfused, # hypoperfused vs hypoperfused + GW2580.
