# Supplementary material for: Inhibiting CSF1R alleviates cerebrovascular white matter disease and cognitive impairment
Source: Glia. 2023 Nov 1;72(2):375–95. doi: 10.1002/glia.24481 (PMC10952452; doi:10.1002/glia.24481)
Supplement: Supplementary file 2 — Table S1: Supplementary Table 1. [file GLIA-72-375-s003.docx]

**Supplementary Tables**

|  | **Gene** | **Forward (5’-3’)** | **Reverse (5’-3’)** | **Cycling conditions** |
| --- | --- | --- | --- | --- |
| **Mouse** | *18S* | cccagtaagtgcgggtcat | ccgagggcctcactaaacc | 60°C for 20 s + 72°C for 30 s |
|  | *Csf1r* | gaaggaaggccgaggctatg | gagaagccactgtccctgc |  |
|  | *Aif1* | tggaggggatcaacaagcaa | tccatttccattcagatcaaatcc |  |
|  | *Cd68* | tcagctaaactcgctcaatc | tccagcctgttgtaactgag | 62.5°C for 30 s |
| **Human** | *18S* | ggccctgtaattggaatgagtc | ccaagatccaactacgagctt | 65°C for 30 s |
|  | *CSF1R* | tttggggctagacagactgg | cctgagctgagtgtggtctg |  |
|  | *AIF1* | gggagacgttcagctaccc | ggcttttccttttctctcgct |  |
|  | *CD68* | gcagaaagcaataagcacca | aaagtttctcctgccccagt | 60°C for 30 s |

**Supplementary Table 1: Custom-designed primers and primer-specific cycling conditions for qPCR.**

**Supplementary Tables 2, 3 and 4 in separate file on-line**
